# Metabolic impairments associated with type 2 diabetes mellitus and the potential effects of exercise therapy: An exploratory randomized trial based on untargeted metabolomics

**DOI:** 10.1371/journal.pone.0300593

**Published:** 2024-03-22

**Authors:** Furong Zhang, Xixi Chen, Mingxiao Yang, Xiaoyu Shen, Yiliang Wang, Dongling Zhong, Fang Zeng, Rongjiang Jin

**Affiliations:** 1 College of Health Preservation and Rehabilitation, Chengdu University of Traditional Chinese Medicine, Chengdu, Sichuan, China; 2 College of Acupuncture and Tuina, Chengdu University of Traditional Chinese Medicine, Chengdu, Sichuan, China; 3 Department of Medicine, Memorial Sloan Kettering Cancer Center, New York, NY, United States of America; 4 Second Affiliated Hospital of Chengdu Medical College/Nuclear Industry 416 Hospital, Chengdu, Sichuan, China; 5 Chongqing University Three Gorges Hospital, Chongqing, China; 6 Acupuncture-Brain Science Research Center, Chengdu University of Traditional Chinese Medicine, Chengdu, Sichuan, China; University of Rwanda College of Medicine and Health Sciences, RWANDA

## Abstract

**Background:**

Type 2 diabetes mellitus (T2DM) is a common condition that is characterized by metabolic impairments. Exercise therapy has proven effective in improving the physiological and psychological states of patients with T2DM; however, the influence of different exercise modalities on metabolic profiles is not fully understood. This study first aimed to investigate the metabolic changes associated with T2DM among patients and then to evaluate the potential physiological effects of different exercise modalities (Tai Chi and brisk walking) on their metabolic profiles.

**Methods:**

This study included 20 T2DM patients and 11 healthy subjects. Patients were randomly allocated to either the Tai Chi or walking group to perform *Dijia* simplified 24-form Tai Chi or brisk walking (80–100 m/min), with 90 minutes each time, three times per week for 12 weeks, for a total of 36 sessions. The healthy group maintained daily living habits without intervention. Glycemic tests were conducted at the baseline and after 12 weeks. Serum and urine samples were collected for untargeted metabolomic analyses at baseline and 12 weeks to examine the differential metabolic profiles between T2DM and healthy subjects, and the metabolic alterations of T2DM patients before and after exercise therapy.

**Results:**

Compared to the healthy group, T2DM patients exhibited metabolic disturbances in carbohydrates (fructose, mannose, galactose, glycolysis/gluconeogenesis), lipids (inositol phosphate), and amino acids (arginine, proline, cysteine, methionine, valine, leucine, and isoleucine) metabolism, including 20 differential metabolites in the serum and six in the urine. After exercise, the glycemic results showed insignificant changes. However, patients who practiced Tai Chi showed significant improvements in their post-treatment metabolic profiles compared to baseline, with nine serum and six urine metabolites, including branch-chained amino acids (BCAAs); while those in the walking group had significantly altered nine serum and four urine metabolites concerning steroid hormone biosynthesis and arachidonic acid metabolism compared to baseline.

**Conclusion:**

T2DM patients displayed impaired carbohydrate, lipid, and amino acid metabolism, and exercise therapy improved their metabolic health. Different modalities may act through different pathways. Tai Chi may improve disrupted BCAAs metabolism, whereas brisk walking mainly regulates steroid hormone biosynthesis and arachidonic acid metabolism.

## Introduction

Type 2 diabetes mellitus (T2DM), a typical metabolic disorder of global concern, accounts for over 90% of all types of diabetes [1]. The prevalence of T2DM is estimated to be 12.2% by 2045, reaching 783 million with a global health expenditure of USD 966 billion by 2021 according to the IDF Diabetes Atlas (10th edition) [2]. China reached a national prevalence of 12.8%, with nearly 130 million diabetic patients in 2017 [3].

T2DM is a metabolic disorder characterized by hyperglycemia and altered lipid metabolism closely associated with metabolic impairment [4] which can be detected by metabolomics. Metabolomics systematically identifies all small molecules called metabolites in biological fluids and tissues using nuclear magnetic resonance spectroscopy (NMR) or mass spectrometry (MS). Modern high-throughput metabolomics have shed light on the roles of metabolites (amino acids, bile acids, and lipids) in complex physiological processes, such as insulin sensitivity regulation [5]. For instance, saturated fatty acids, such as palmitic acid, directly impair the insulin signaling pathway by activating proinflammatory pathways, including toll-like receptors (TLRs), especially TLR4 [6]. Products from increased lipolysis due to insulin-mediated suppression impairment indirectly upregulates the substrate flux, leading to more glucose production [7]. Glycerol, the essential gluconeogenic precursor, increases gluconeogenesis in non-insulin-dependent diabetes [8], and free fatty acid activated TLR4 or TLR2 to induce adipose tissue macrophage, further leading to inflammatory cytokines and insulin resistance [9]. Acetyl-CoA and palmitic acid or myristic acid alter protein functions by modifying post-translational acetylation and palmitoylation [5]. A metabolic pattern is referred to a specific metabolic profile closely associated with or even can diagnose a certain condition/disease, where the robust metabolic biomarkers come from. Recent studies have shown that T2DM patients exhibit an altered metabolic pattern related to decreased short-chain fatty acids (SCFAs), increased branched-chain amino acids (BCAAs), and perturbated bile acid metabolism [10,11], which is colsey associated with T2DM development. Transplanting detrimental microbiota from T2DM donors to germ-free mice can induce early symptoms and pathological changes in T2DM due to the microbiota-derived metabolites [12,13], while reversing metabolic abnormalities can attenuate these clinical symptoms and signs like insulin resistance and systematic chronic inflammation. Given the significance of metabolic health in the pathophysiology of T2DM, examining the metabolic changes associated with T2DM is important to better understand the disease and explore novel therapeutics.

Lack of exercise is considered a major risk factor for T2DM. Exercise therapy, as a cost-effective approach, is increasingly recognized and applied in T2DM prevention and management [14,15]. The physiological effects of exercise improve metabolic health by adapting almost all body tissues including muscle, adipose tissue, liver [16]. However, due to the complex and heterogeneous pathophysiology of T2DM, coupled with the integrative biological mechanisms of exercise, the impact of different exercise modalities on the metabolic health of T2DM patients remains elusive. Considering that metabolites are the end products of cellular regulatory processes [17], the metabolome, the collection of all metabolites, reflects what has occurred in the organism. Metabolomics is a promising approach for exploring complex interactions [18]. Metabolomics has been increasingly applied to study exercise physiology and exercise-associated metabolism [19]. In China, Tai Chi and brisk walking are popular exercise modalities among the elderly Chinese. Tai Chi, a mind-body practice that integrates meditation and physical movement, has been employed in clinical rehabilitation of chronic conditions. Its effect has been validated in several chronic diseases including metabolic syndrome [20], fibromyalgia [21], knee osteoarthritis [22], Parkinson’s disease [23], and insomnia after breast cancer [24], etc. Our previous clinical study has demonstrated the hypoglycemic effect of Tai Chi [25], yet its influence on metabolic profiles has not been investigated. Brisk walking, another common exercise modality, was reported to reduce risk of T2DM [26,27]. Although aerobic exercises benefit patients with T2DM, they seem work in different ways regarding metabolic homeostasis. As an ideal tool for exercise-related researches, metabolomics has been validated its feasibility to explore the potential mechanisms of Tai Chi among patients [28,29].

Therefore, this study was to investigate the metabolic changes associated with T2DM among patients and to further explore the potential mechanism of different aerobic exercise modalities including Tai Chi on T2DM patients in the perspective of metabolomics.

## Materials and methods

### Ethics approval and trial registration

This study was approved by the Ethics Review Committee of the Hospital of Chengdu University of Traditional Chinese Medicine (2018KL-028) and the study protocol was registered on the Chinese Clinical Trial Registry platform (http://www.chictr.org.cn/index.aspx) with an identifier of ChiCTR1800018440. All the procedures were designed and conducted in accordance with the principles of the Declaration of Helsinki. Written informed consent was obtained from all participants. We used the CONSORT checklist when writing our report [30].

### Study design

**Fig 1:**
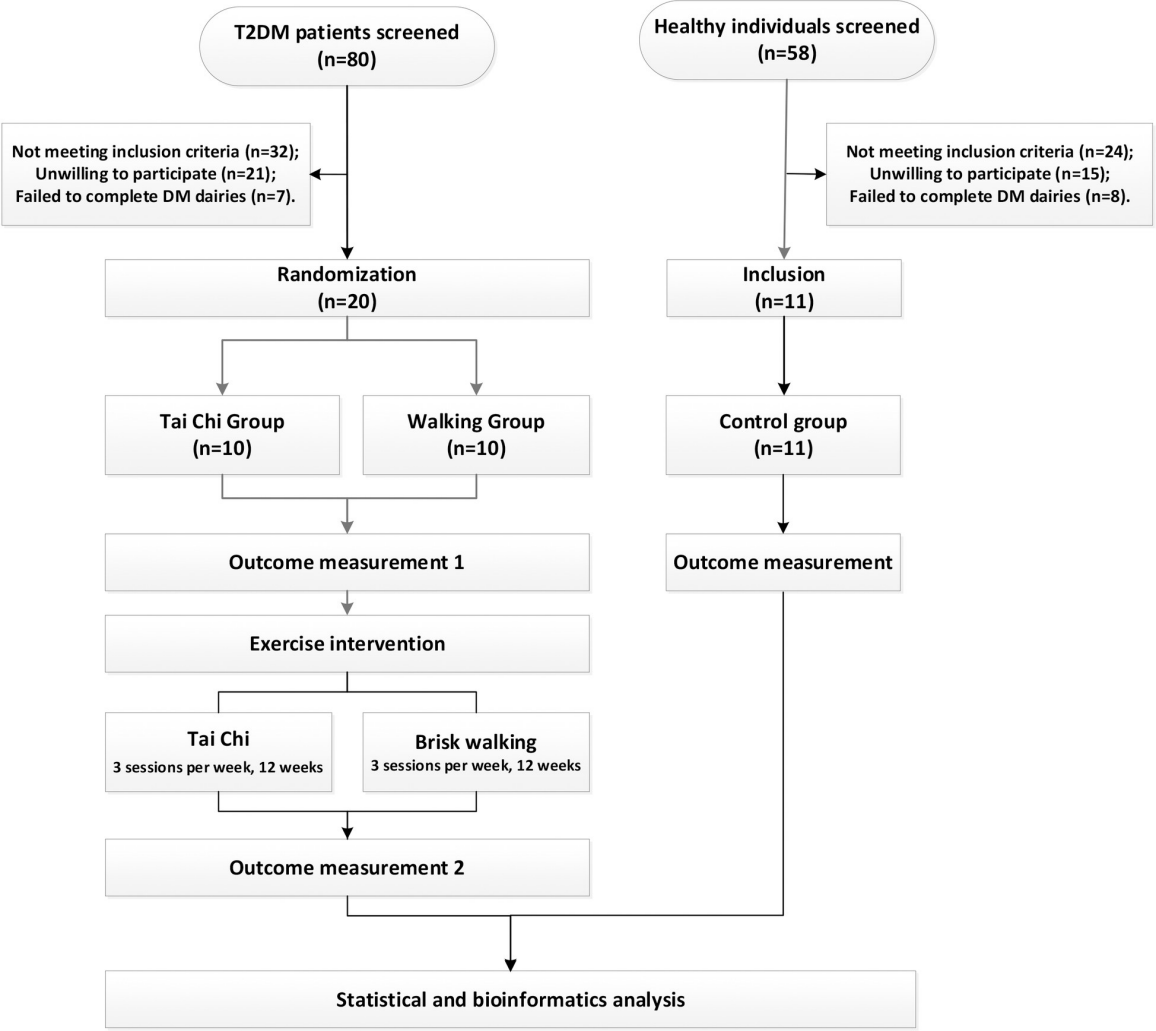
Study flowchart. The workflow of this study was demonstrated in this flowchart.

This study was an open-label, three-arm, randomized controlled trial conducted at the Hospital of Chengdu University of Traditional Chinese Medicine and the Third Affiliated Hospital of Chengdu University of Traditional Chinese Medicine, Sichuan, China. A total of 20 T2DM patients and 11 healthy individuals were recruited. The eligible T2DM patients were randomized in a ratio of 1:1 by using a list of computer-generated randomization numbers to either the Tai Chi or walking group, with 10 patients in each group. Allocations were concealed in opaque envelopes, and they were unpacked when eligible participants were recruited by another researcher. The patients underwent 36 sessions of exercise intervention over 12 weeks. The primary outcomes were: changes in fasting blood glucose (FBG) and hemoglobinA1c (HbA1c) levels from baseline to the end of the intervention. Secondary outcomes included changes in serum and urine metabolomic profiles, and adverse events such as falls, bruises, bone fractures etc. The flowchart of this study is shown in Fig 1.

Throughout the duration of the study, a series of protocol deviations arose as a result of the unprecedented COVID-19 outbreak. The specific instances of these deviations are outlined below:

Modification of sample size: The sample size for each experimental group was revised, necessitating a reduction from 15 to 10 cases per group. This adjustment was prompted by logistical constraints posed by the pandemic, which impacted participant recruitment and retention.

Alteration of Secondary Outcomes: Several changes were made to the secondary outcome measures due to pandemic-related challenges. The measurement of gastrin was omitted due to inherent difficulties in procuring an adequate quantity of biofluid samples. Additionally, the inclusion of SF-12 and RPE data was curtailed. This decision was predicated on the substantial time and energy demands associated with the completion of daily diaries. Furthermore, certain participants encountered difficulties with the electronic diary format, leading to incomplete entries.

### Study participants

The participants recruitment took place from October 2018 to January 2020. All study participants were recruited from the Teaching Hospital of Chengdu University of Traditional Chinese Medicine and the Third Affiliated Hospital of Chengdu University of Traditional Chinese Medicine through clinic visits, physician referrals, and electronic advertisements via the WeChat app. Healthy participants were recruited from local communities.

T2DM patients and healthy subjects were included if they met all the following criteria.

#### T2DM patients

1. **Diagnostic criteria**
  The patients were diagnosed with T2DM according to the 2013 Chinese Guidelines for the Prevention and Treatment of Diabetes (fasting glucose > = 7.0 mmol/L and HbA1c > = 6.5%).
2. **Inclusion criteria**
  - male or female, between ages 45 to 75 years
  - of Han ethnicity, and have lived in Chengdu city for over 5 years
  - no exercise regularly within the past three months and not practice Tai Chi for past two months
  - no recent history of gastrointestinal disorders
  - no administration of antibiotics, prebiotics, or probiotics in the past two months
  - willingness to participate in this study and provide written confirmed consent
3. **Exclusion criteria**
  - with heavy smoking, alcohol, or drug abuse
  - women during pregnancy or lactation
  - with severe diabetic complications of the heart, brain, kidney, peripheral nerves, and retina
  - with any history of severe cardiovascular or cerebrovascular diseases, tumors, autoimmune disease, chronic inflammation, recent infection, or surgery
  - with severe hepatic or renal insufficiency or severe hypertension (systolic blood pressure (SBP) > 160 mmHg, diastolic blood pressure (DBP) > 95 mmHg)
  - hyperglycemia secondary to conditions like hyperthyroidism, Cushing syndrome, etc.
  - with severe skeletomuscular diseases or motor dysfunctions unsuitable for exercise
  - participating in other clinical trials

#### Healthy subjects

Healthy individuals of similar age, gender, ethnicity, and body mass index (BMI) to that of T2DM patients were recruited as controls. The healthy subjects do not have organic or functional disorders and do not have regular exercise within the past three months. Additionally, those smokers, alcoholics, and women during pregnancy or lactation were excluded.

### Interventions

#### Tai Chi intervention

Participants in the Tai Chi group were taught simplified 24-form Tai Chi by a certified coach (with over 2 years of experience) in a one-to-one manner. The participants underwent exams for qualification based on the accuracy, coordination, and continuity of their movements. Those who had successfully passed the test qualified to participate in the 12-week Tai Chi training program. The program consisted of 36 sessions, with three sessions per week for 12 weeks, and each session lasted 90 minutes, including a 20-minutes warm-up, 60-minutes Tai Chi practice, and 10-minutes cool-down. Considering that most participants were elderly patients with diabetes, *Gaojia* Tai Chi was selected for this study. It consists of the same 24 movements as simplified Tai Chi but is characterized by a lifted gravity center and an over 150° knee flexion of the practitioner, which is less energy-consuming when compared to other postures such as *Dijia* (low posture, knee flexion < 120°), which makes it safer and more suitable for the elderly to practice.

#### Walking intervention

Participants in the walking group participated in a brisk walking program (20-minutes warm-up, 60-minutes walking and 10-minutes cool-down) of the same duration and frequency as the Tai Chi group, which was based on the results of metabolic equivalent (MET) conversion. The average energy consumption of the elderly practicing *Gaojia* Tai Chi was 2.63±0.31 METs [28], and the 3–5 minutes rest interval accounts for approximately 1/7–1/5 of the total energy consumption [29]; and the energy consumption of brisk walking (80–100 m/min) is about 4–5.5 METs [30].

#### Control

Healthy subjects in the control group received no extra intervention but to maintain their routine living and eating habits, complete the examination, and collect information on time.

All participants received the same health education and completed a self-reported diabetes diary, which included items on daily fingertip blood glucose (three times per day), food categories, medication, exercise, and mood. This was to help them develop a habit of T2DM self-management. The participants were required to have good compliance by attending at least 90% of the prescribed exercise sessions. All participants wore smart bracelets (Honor 3, NYX-B10, Huawei Device Co. Ltd., China) provided by the research group to monitor their heart rate during exercise, thus monitoring the intensity during exercise.

### Measurements

#### Clinical outcomes

Basic demographic information and physiological data on blood sugar and lipids (HbA1c, fasting plasma glucose, total cholesterol, ALT, AST, serum creatinine and serum uric acid) were obtained at baseline. Changes in FBG levels and HbA1c levels in superficial venous blood were detected from baseline to the end of the intervention. Safety was measured by adverse events such as falls, faint, bruises, etc.

#### Biological sample collection, storage, and preparation

Biospecimens of T2DM patients in the Tai Chi and walking groups were collected at baseline and post-intervention, whereas those of healthy controls were collected at baseline. After overnight fasting for approximately 10 hours, the morning midstream urine samples (> = 20 ml) were collected into standard urine sample cups, and fasting venous blood samples (> = 5 ml)were collected between 8 a.m. and 10 a.m. in the TCM Hospital of Sichuan Province/Teaching Hospital of Chengdu University of TCM, China. The venous whole blood samples were collected in EDTA-anticoagulated vials for fasting blood glucose and HbA1c.The biochemical examinations were conducted by the clinical laboratory department of the Hospital of Chengdu University of Traditional Chinese Medicine. Urine and blood samples for untargeted metabolomics were centrifuged at 3000 rpm at 4°C for 10 minutes, and then aliquoted into 1.5mL tubes, and stored at -80°C until analysis. Biofluid samples for LC-MS untargeted metabolomics were prepared and pretreated according to the standard operating procedure. After thawing at 4°C, 100μL samples and 300μL precooled methanol (1:3, v:v) were added, followed by vortexing for 30 seconds and centrifuged at 15,000 rpm for 10 minutes at 4°C. The supernatant was extracted for untargeted metabolomic analysis. The untargeted metabolomics were conducted by BGI company.

#### Untargeted metabolomics profiling

Untargeted metabolomic profiles were generated using a 2777C Ultra-Performance Liquid Chromatography (UPLC) system (Waters, UK) and a Xevo G2-XS Quadrupole Time-of-Flight Mass Spectrometry (Q-TOF) (Waters, UK). All samples were acquired using an LC-MS system followed by machine orders. All chromatographic separations were performed using a UPLC system. The ACQUITY UPLC HSS T3 column (100mm*2.1mm, 1.8μm, Waters, UK) was applied for the reversed phase separation, with the column oven at 50°C and flow rate of 0.4mL/min. The mobile phases were 0.1% formic acid in water (solvent A) and 0.1% formic acid in methanol (solvent B). The Gradient elution conditions were set as follows: 0–2 minutes, 100% phase A; 2–11 minutes, 0% to 100% B; 11–13 minutes, 100% B; 13–15 minutes, 0% to 100% A. The HSS T3 column was injected with 5μl of each sample. Q-TOF was used to detect the metabolites eluted from the column. The positive ion mode employed capillary and sampling cone voltages of 3.0kV and 40.0V, respectively, whereas the negative mode was set at 2.0kV and 40.0V, respectively. The mass spectrometry data were acquired in the Centroid MSE mode. The TOF mass range was from 50 to 1200Da and the scan time was 0.2 seconds. For MS/MS detection, all precursors were fragmented using 20-40eV, and the scan time was 0.2 seconds. During the acquisition, the LE signal was acquired every three seconds to calibrate the mass accuracy. Furthermore, to monitor the stability, repeatability, and reliability of the system, quality control (QC) samples were prepared by mixing equal volumes of each experimental sample. Ten QCs were continuously injected at the beginning and then at regular intervals of 10 samples throughout the analytical run to monitor the retention time (RT/min) shift and signal variations.

#### Untargeted metabolome data processing and metabolite identification

Progenesis QI (version 2.2, Waters, UK, hereinafter referred to as QI) and the metaX package [31] in R Software were used for metabolome data processing. Raw MS data in both positive and negative ion modes were imported into the QI for peak alignment, peak extraction, normalization, deconvolution, and compound identification. MetaX was used to preprocess the data. The K-nearest neighbor (KNN) method was applied for missing value imputation and low-quality ion removal (ion missing > 50% of the QC samples or > 80% of the experimental samples), and the quality control-based robust LOESS signal correction (QC-RSC) [32] method was employed to correct signal drift, after which, the corrected data were filtered by removing those with a relative standard deviation (RSD) > 30% in the QC samples. The filtered data were used for further statistical analyses.

QI was used to identify and annotate the metabolites. Metabolite identification is based on retention time, the mass spectrum, and the marker ions. Each reported metabolite was matched in the Human Metabolome Database (HMDB, https://hmdb.ca/). The screening criteria for differential metabolites were based on the variable importance plot (VIP) value of the first two principal components of partial least squares discriminant analysis (PLS-DA), together with the fold-change (FC) and p value. Those meeting all the following criteria simultaneously were identified as differential metabolites: (1) VIP value > = 1.0; (2) FC value > = 1.2 or < = 0.8333; (3) p value < = 0.05. Metabolic pathway analysis was performed using the Kyoto Encyclopedia of Genes and Genomes (KEGG) database (https://www.kegg.jp/). All identifications were further confirmed manually by the identification score, isotope similarity score, and biological background.

### Statistical analyses

For clinical data, categorical data were expressed as frequencies or constitution ratios, and quantitative data were presented as quartiles. Inter-group comparison of quantitative data was performed using the t-test or Mann-Whitney U test, and intragroup comparisons were performed using the paired t-test or Wilcoxon rank sum test. Pearson’s Chi-square test was used for inter-group comparisons of categorical data. The test level α was 0.05, and p value < 0.05 (two-tail) was considered statistically significant. GraphPad Prism software (version 8.0) was used for the clinical data analysis (https://www.graphpad.com/).

Both univariate and multivariate analyses were used to analyze metabolome data. Normalized datasets were unit variance scaled before multivariate analysis. Paired t test (p < 0.05) and FC value > = 1.2 or < = 0.8333 were used for univariate analysis, and the results are presented in the volcano plot. Multivariate analysis, including unsupervised principal component analysis (PCA) and supervised partial least squares discriminant analysis (PLS-DA) were used which reduced the dimensionality and enabled the visualization of the separation of different groups, and further identified potential biomarkers. PCA of the unsupervised model was employed to analyze the differences and inter- and intra-group variation, whereas supervised PLS-DA enabled clear discrimination between the two groups. R2 and Q2 values were obtained from 200 random permutations in the cross-validation analysis to indicate the predictive capacity of the PLS-DA model.

## Results

### Demographic information

In this study, 20 eligible T2DM patients and 11 healthy subjects were enrolled. Apart from FBG and HbA1c, other parameters showed no significant differences between T2DM patients and healthy controls. Besides, there were no statistical differences on duration of illness, comorbidities and concomitant medication between T2DM patients in Tai Chi and walking group. No exercise-related adverse events were detected during study. The details of the study’s participant demographics are displayed in Table 1.

**Table 1:** Demographic information.

Characteristics	T2DM (n = 20)			Control (n = 11)	p value
	Tai Chi (n = 10)	Walking (n = 10)	Total		
Male sex, n(%)	6(60%)	5(50%)	11(55%)	7(64%)	0.98
Age (years), median (Q1, Q3)	64.50(62.25, 70.25)	66.50(61.75, 71.50)	64.50(62.25, 70.25)	60.00(57.00, 64.00)	0.05
Illness duration(years), median (Q1, Q3)	7.00(3.00, 17.30)	7.00(3.75, 20.00)	-	-	0.46
Comorbidities, n (%)					
Hypertension	2(20%)	1(10%)	-	-	>0.99
Hyperlipidemia	2(20%)	1(10%)	-	-	>0.99
Concomitant medication, n (%)					
Metformin	4(40%)	5(50%)	-	-	>0.99
Sulfonylurea	2(20%)	3(30%)	-	-	>0.99
Antihypertensive agents	2(20%)	1(10%)	-	-	>0.99
BMI (kg/m^2^), median (Q1, Q3)	23.09(22.17, 27.36)	23.37(19.94, 27.58)	23.19(21.53, 27.38)	22.51(20.82, 24.38)	0.30
FBG (mmol/L), median (Q1, Q3)	7.94(6.51, 9.30)	6.64(5.87, 8.25)	7.73(6.34, 8.76)	5.08(4.74, 5.18)	< 0.001**
HbA1c (%), median (Q1, Q3)	6.60(6.15, 8.03)	6.25(5.80, 7.65)	6.40(5.93, 7.75)	5.30(5.10, 5.50)	< 0.001**
Total Cholesterol (mmol/L), median (Q1, Q3)	4.74(4.44, 5.20)	4.43(3.27, 5.13)	4.64(4.08, 5.10)	4.74(4.06, 5.32)	0.72
ALT (unit/L), median (Q1, Q3)	23.00(14.25, 31.75)	20.50(14.75,25)	21.50(15.00, 27.00)	18.00(14.00, 22.00)	0.31
AST (unit/L), median (Q1, Q3)	22.50(18.25, 27.75)	22.00(19.75, 22.75)	22.00(19.25, 24.75)	21.00(20.00, 28.00)	0.75
Serum creatine (μmol/L), median (Q1, Q3)	64.10(56.88, 78.28)	63.70(50.68, 72.68)	63.7(56.83, 75.70)	61.00(55.50, 79.00)	0.73
Serum uric acid (μmol/L), median (Q1, Q3)	377.50(308.50, 464.30)	366.50(306.30, 414.80)	366.50(309.00, 418.30)	311.00(283.00, 392.00)	0.24

median (Q1, Q3), Q1, first quartile; Q3, third quartile

**p<0.01 represents significant difference.

### T2DM patients and healthy controls were of different metabolic profiles

**Fig 2:**
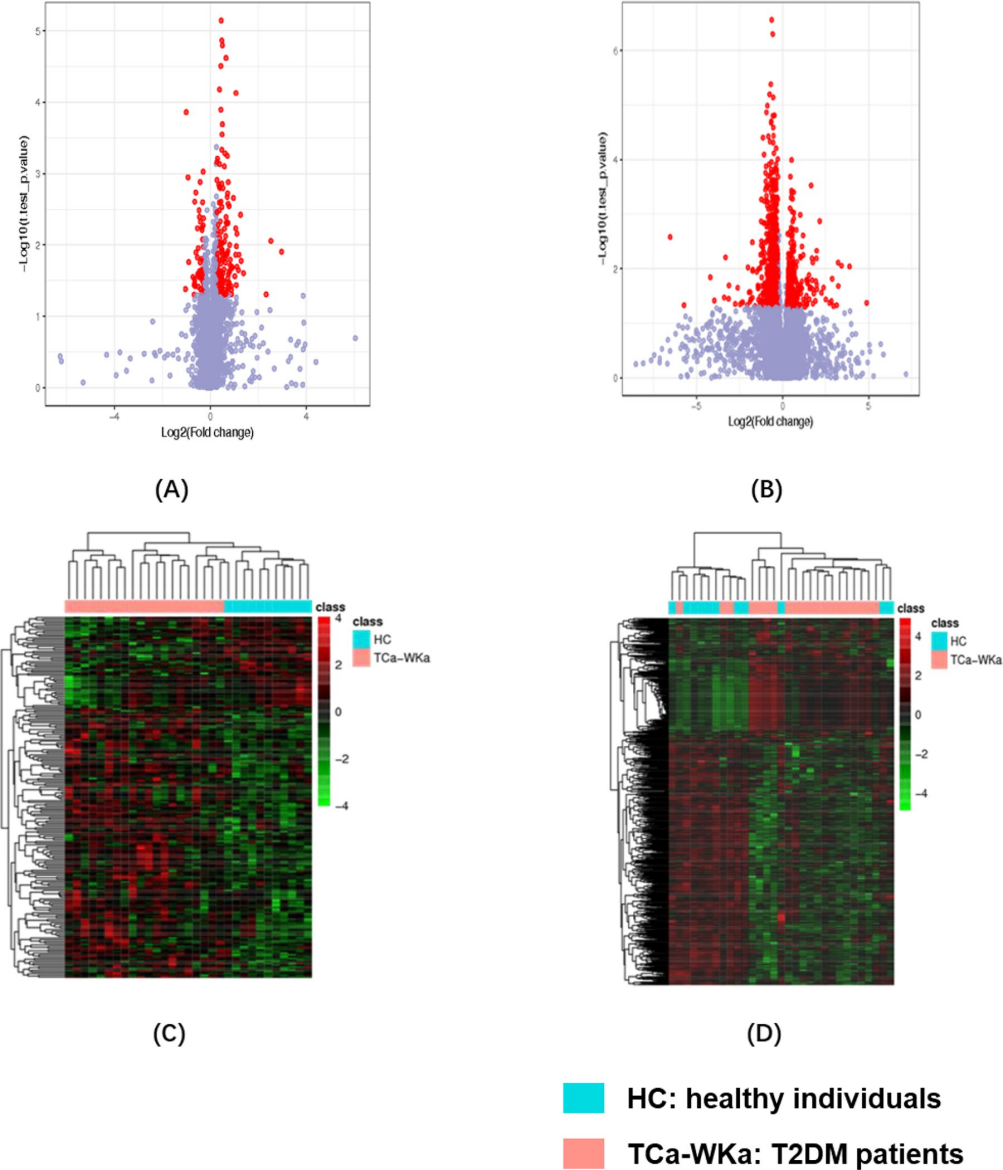
Univariate statistical analysis volcano plots (fold change value > = 1.2 or < = 0.8333, and t test p-value <0.05) and heatmaps for differential metabolites between the T2DM patients and healthy individuals in positive ion mode. (A) volcano plot in serum;(B) volcano plot in urine; (C) heatmap in serum; (D) heatmap in urine. In volcano plots, each dot represents a metabolite, those in red are potential differential metabolites based on univariate statistical analysis while light purple ones are not eligible.

**Fig 3:**
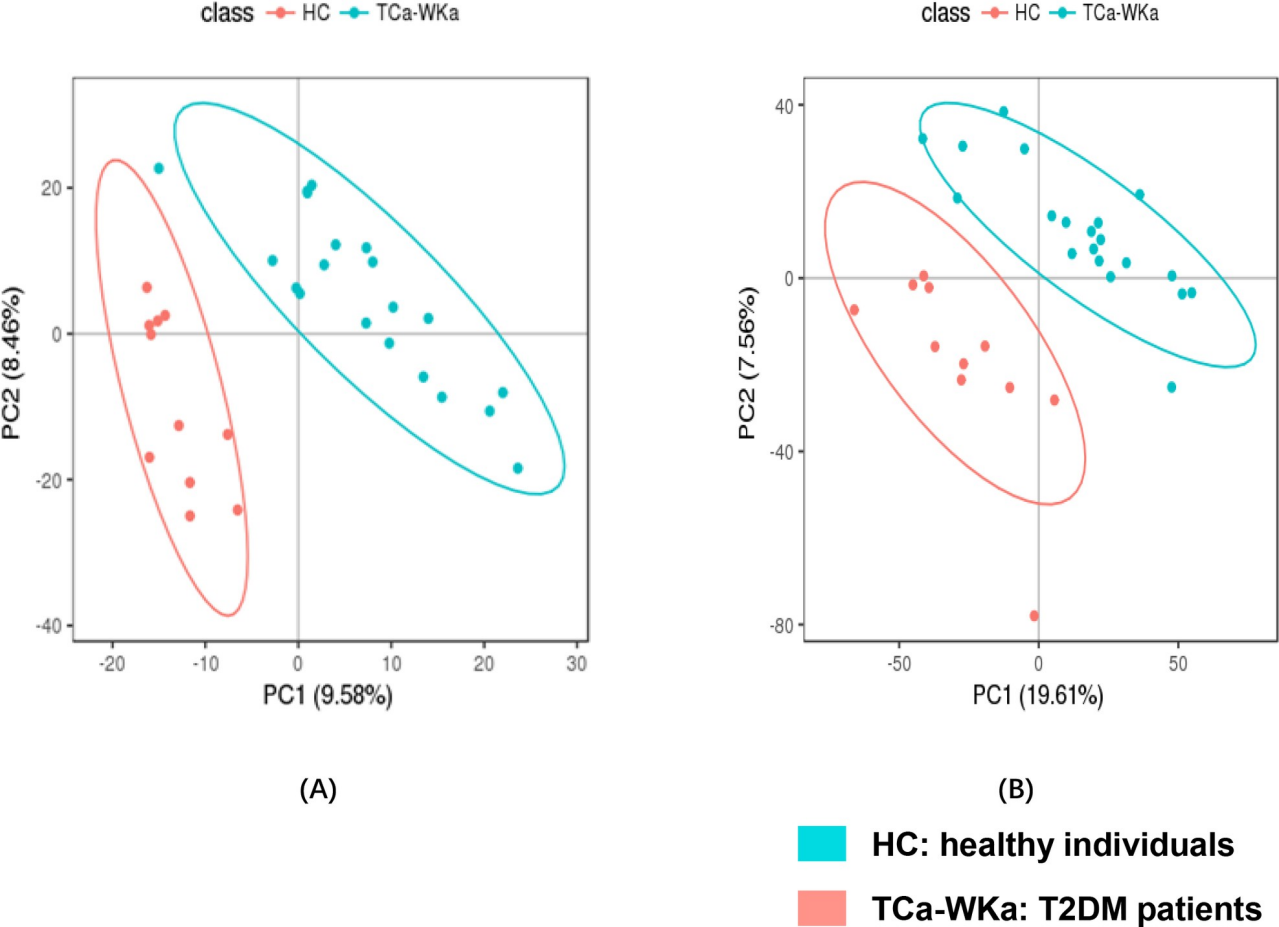
Multivariate statistical analysis PLS-DA for identifying differential metabolites between T2DM and healthy individuals in positive ion mode. (A) PLS-DA in serum post and pre Tai Chi, R2 = 0.893, Q2 = -0.010; (B) PLS-DA in urine post and pre Tai Chi in positive ion mode, R2 = 0.8454, Q2 = 0.0747.

Metabolomic analysis revealed different metabolic profiles between patients with T2DM and healthy controls. Univariate analysis showed that multiple differential metabolites were detected and visualized using volcano plots and heatmaps (Fig 2). The results of unsupervised PCA are shown in S1 Fig. To identify potential biomarkers between patients with T2DM and healthy controls, the following filtration criteria were applied: VIP value >2.0 of the supervised PLS-DA models, p value <0.05, Student’s t-test, and FC value > = 1.2 or < = 0.8333 (Fig 3). Twenty-six potential biomarkers in the T2DM group were found in serum and urine metabolomics, with one biomarker downregulated and the remaining 25 upregulated in serum; four downregulated and two upregulated in urine. Information on serum and urine biomarkers, including compound ID, changing trend, VIP value, KEGG ID, identification, and corresponding KEGG pathways are displayed in Table 2. These biomarkers are related to the pathways of glycometabolism, lipid metabolism, and amino acid metabolism, which are typical metabolic disorders among patients with diabetes.

**Table 2:** Identified differential metabolites in serum and urine between the T2DM group and healthy control group in positive (+) and negative (-) ion modes.

Sample	No.	Compounds ID	Trend	VIP	KEGG ID	Identification	Pathway
Serum	1+	0.61_203.0532m/z	↑	2.64	C00159	D-mannose	Fructose mannose metabolism; Galactose metabolism
	2+	0.61_203.0532m/z	↑	2.64	C00031	D-glucose	Glycolysis / Gluconeogenesis
	3+	0.61_203.0532m/z	↑	2.64	C00984	D-galactose	Galactose metabolism
	4+	0.61_203.0532m/z	↑	2.64	C00137	Myo-inositol	Galactose metabolism; Inositol phosphate metabolism
	5+	0.61_203.0532m/z	↑	2.64	C19891	D-chiro-inositol	Inositol phosphate metabolism
	6+	0.61_203.0532m/z	↑	2.64	C06153	Scyllo-inositol	
	7+	4.32_130.0497m/z	↑	2.58	C01879	Pyroglutamic acid	Glutathione metabolism
	8+	4.32_130.0497m/z	↑	2.58	C05938	L-4-hydroxyglutamate semialdehyde	Arginine and proline metabolism
	9+	4.32_130.0497m/z	↑	2.58	C00217	D-glutamic acid	D-Glutamine and D-glutamate metabolism
	10+	4.32_130.0497m/z	↑	2.58	C00979	O-acetylserine	Cysteine and methionine metabolism
	11+	0.68_130.0861m/z	↓	2.49	C00408	L-pipecolic acid	Lysine degradation
	12+	0.59_125.0209m/z	↑	2.02	C00349	2-methyl-3-oxopropanoic acid	Valine, leucine and isoleucine degradation
	13+	0.59_125.0209m/z	↑	2.02	C06002	(S)-methylmalonic acid semialdehyde	Valine, leucine and isoleucine degradation
	14+	0.59_125.0209m/z	↑	2.02	C00109	2-ketobutyric acid	Glycine, serine and threonine metabolism
	15+	0.59_125.0209m/z	↑	2.02	C00232	Succinic acid semialdehyde	Alanine, aspartate and glutamate metabolism
	16+	0.59_125.0209m/z	↑	2.02	C00164	Acetoacetic acid	Synthesis and degradation of ketone bodies; Valine, leucine and isoleucine degradation
	17-	6.52_511.2525m/z	↑	3.70	C11136	Etiocholanolone glucuronide	Steroid hormone biosynthesis
	18-	4.31_145.0615m/z	↑	3.11	C00064	L-glutamine	Arginine biosynthesis
	19-	4.31_263.1031m/z	↑	2.64	C04148	Phenylacetylglutamine	Phenylalanine metabolism
	20-	9.63_293.2117m/z	↑	2.49	C14766	9-OxoODE	Linoleic acid metabolism
Urine	1+	3.51_229.0723m/z	↓	2.26	C06212	N-methylserotonin	Tryptophan metabolism
	2+	3.51_229.0723m/z	↓	2.26	C05659	5-methoxytryptamine	Tryptophan metabolism
	3-	5.37_437.0540m/z	↑	2.46	C04352	4-phosphopantothenoylcysteine	Pantothenate and CoA biosynthesis
	4-	6.44_453.2843m/z	↓	2.34	C00695	Cholic acid	Primary bile acid biosynthesis
	5-	3.72_206.0454m/z	↑	2.32	C05637	Quinoline-4,8-diol	Tryptophan metabolism
	6-	3.43_228.0875m/z	↓	2.05	C00788	Epinephrine	Tyrosine metabolism

T2DM group VS healthy control group in serum and urine metabolomics: p < 0.05, VIP > 2, FC > = 1.2 or < = 0.8333.+: positive ion mode; -: negative ion mode; ↑: up-regulated; ↓: down-regulated.

### Exercise-induced metabolomic alteration of T2DM patients; yet the modes of action seem differ between Tai Chi and brisk walking in perspective of metabolomics

**Fig 4:**
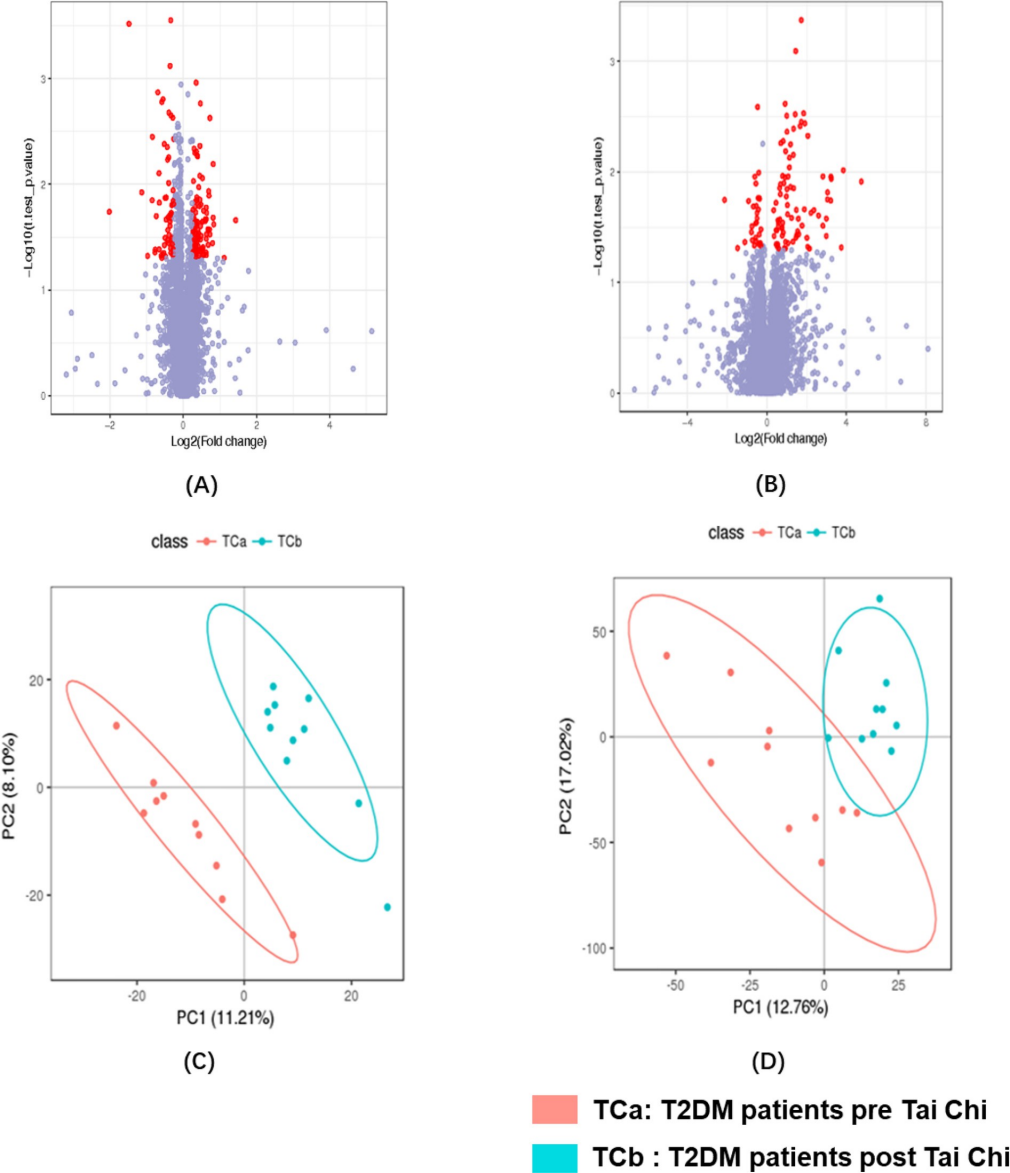
Univariate volcano plots and multivariate PLS-DA in serum and urine metabolomics change of T2DM post and pre Tai Chi in positive ion mode. Volcano plots for differential metabolites post and pre Tai Chi. (A) volcano plot in serum post and pre Tai Chi; (B) volcano plot in urine post and pre Tai Chi. PLS-DA for identifying differential metabolites post and pre Tai Chi. (C) PLS-DA in serum post and pre Tai Chi, R2 = 0.957, Q2 = -0.246; (D) PLS-DA in urine post and pre Tai Chi, R2 = 0.8007, Q2 = -0.9697.

After a 12-week 36-session exercise intervention, T2DM patients in both Tai Chi and walking groups underwent metabolomic alterations in serum and urine. Glycemic outcomes showed no statistically significant improvement in any inter or intra group comparisons (S1 Table). Results of unsupervised PCA post and pre Tai Chi and walking were shown in S2 Fig. Volcano plots and PLS-DA were demonstrated in Figs 4 and S3.

The differential metabolites before and after exercise were filtered using the criteria mentioned above. Fifteen potential biomarkers were found after Tai Chi exercise in serum and urine metabolomics, respectively, with three biomarkers downregulated and four upregulated in serum and one downregulated and five upregulated in urine. Information on biomarkers post-Tai Chi and walking were displayed in Table 3 and S2 Table, respectively. Biomarkers post-Tai Chi are mainly involved in sphingolipid metabolism, primary bile acid biosynthesis, and metabolism of xenobiotics by cytochrome P450, while those after walking are related to pathways including steroid hormone biosynthesis, phenylalanine metabolism, arachidonic acid metabolism, lipid metabolism, and amino acid metabolism, which are closely related to diabetes.

**Table 3:** Identified differential metabolites in serum of T2DM patients post and pre Tai Chi in positive (+) and negative (-) ion modes.

Sample	No.	Compound ID	Trend	VIP	KEGG ID	Identification	Pathway
Serum	1+	7.72_448.3430m/z	↓	2.03	C03640	LysoSM(d18:1)	Sphingolipid metabolism
	2-	0.68_267.0725m/z	↑	3.76	C00294	Inosine	Purine metabolism
	3-	0.61_124.0073m/z	↓	2.68	C00245	Taurine	Primary bile acid biosynthesis
	4-	9.63_293.2117m/z	↓	2.50	C14766	9-OxoODE	Linoleic acid metabolism
	5-	8.68_380.2565m/z	↓	2.48	C01120	Sphinganine 1-phosphate	Sphingolipid metabolism
	6-	8.21_451.3024m/z	↑	2.45	C17332	7alpha,25-Dihydroxy-4-cholesten-3-one	Primary bile acid biosynthesis
	7-	8.21_451.3024m/z	↑	2.45	C01673	Calcitriol	Steroid biosynthesis
	8+	0.59_125.0209m/z	↓	1.83	C00349	2-Methyl-3-oxopropanoic acid	Valine, leucine and isoleucine degradation
	9+	0.59_125.0209m/z	↓	1.83	C06002	(S)-Methylmalonic acid semialdehyde	Valine, leucine and isoleucine degradation
Urine	1+	2.43_153.0789n	↑	3.11	C03758	Dopamine	Tyrosine metabolism
	2+	2.43_153.0789n	↑	3.11	C16666	Vanillylamine	Phenylalanine metabolism
	3+	3.73_212.1283m/z	↑	2.93	C07056	Isoproterenol	Adrenergic signaling in cardiomyocytes
	4+	4.74_366.0503m/z	↑	2.28	C14864	S-(2-Chloroacetyl) glutathione	Metabolism of xenobiotics by cytochrome P450
	5+	4.74_366.0503m/z	↑	2.28	C14865	2-(S-Glutathionyl) acetyl chloride	
	6-	4.52_147.0449m/z	↓	2.75	C05608	(E)-3-(4-Hydroxyphenyl)-2-propenal	

T2DM patients pre and post Tai Chi in serum and urine metabolomics: p < 0.05, VIP > 2 (VIP value of two important metabolites in valine, leucine and isoleucine degradation pathway were btween1.5 to 2), FC > = 1.2 or < = 0.8333.

+: positive ion mode; -: negative ion mode;↑: up-regulated; ↓: down-regulated.

## Discussion

This study is unique in the context of using metabolomics to explore the effects of Tai Chi in human subjects with T2DM. Untargeted metabolomics was employed in this study to investigate the metabolic profile of T2DM patients and explore the effects of a 12-week intervention of Tai Chi and brisk walking on their blood sugar outcomes and metabolic health.

Metabolomic analysis showed that T2DM patients exhibit altered metabolic profiles of carbohydrate, lipid, and amino acid metabolic pathways in both serum and urine compared to healthy individuals. Disturbances in carbohydrate metabolism is the most prominent characteristic of T2DM. Carbohydrates are broken down into simple sugars, including glucose, fructose, and mannose, of which glucose accounts for over 80%, serving as fuel for energy in the human body when transported into cells with the help of insulin. However, the body fails to do so under diabetic conditions, resulting in elevated glucose levels in the blood, which has been confirmed in this and other studies [33]. Perturbated amino acid metabolism has been reported as another emerging key feature of metabolic profiles in T2DM. In this study, the metabolism of BCAAs and aromatic amino acids (AAAs) were disturbed in T2DM patients, which is strongly associated with the prediction and prognosis of diabetes in the Chinese population [34]. BCAAs, such as leucine, isoleucine, and valine are essential amino acids critical for regulating energy homeostasis, nutritional metabolism, gut health, immunity, and disease in both humans and animals [35]. Current evidence suggests that systemic BCAAs and their derivatives act as potential biomarkers of insulin resistance, T2DM, cancer, and cardiovascular diseases [36,37]. AAAs include tryptophan (Trp), phenylalanine, and tyrosine. Accumulating evidence implies that altered metabolism of Trp and its active metabolites play significant roles in the pathogenesis and development of diabetes complications [38,39]. In this study, Trp metabolic intermediates in the serum of the T2DM participants were lower than those in the normal participants, along with results of previous reports [40,41], which correlates to Trp’s slowing gastric emptying with a delayed rise of postprandial glucose [42]. In addition to BCAAs and AAAs, other amino acids also involved in T2DM development, such as glycine, serine, and threonine also changed. The changing trend of several differential metabolites in this study is consistent with previous reports. For instance, acetoacetic acid and phenylalanine have been reported to be positively associated with obesity and diabetes [43,44]. 2-ketobutyric acid in the glycine, serine, and threonine metabolism pathways may be related to impaired glucose tolerance [45]. Diabetes is usually accompanied by aberrated lipid metabolism, another risk factor for cardiovascular disease. Generally, with plentiful glucose, excess acetyl-CoA generated by glycolysis can be converted into lipids such as fatty acids, triglycerides, cholesterol, steroids, and bile salts. However, among patients living with diabetes, especially those with poor glucose control, β-oxidation of free fatty acids is activated as a major energy source to the body, resulting in elevated ketone bodies. In this study, T2DM patients demonstrated increased inositol phosphate metabolism, among which myoinositol is increased in obese and diabetic patients, as reported previously [46]. Increased 9-oxoODE in linoleic acid (LA) metabolism was detected in this study; however, it was significantly diminished in subjects with diabetic macular edema (5). Moreover, LA is associated with a lower risk of T2DM [47]. The inconsistency of elevated dietary LA intake on proinflammatory effects and glucose metabolism may be related to environmental factors besides gender and genetics [48], which calls for further, high-quality studies to reveal the effects of LA metabolism on diabetes.

Sedentary lifestyle is considered a major factor in the development of T2DM. Exercise therapy, including Tai Chi [49–54], has demonstrated great potential in the treatment and rehabilitation of T2DM. Regular exercise has been reported to enhance metabolic health, including insulin sensitivity, lipid metabolism, and body composition [55]. This can lead to substantial and profound changes in organ and tissue metabolism [19]. As the therapeutic application of altered metabolites may affect phenotypes [56], exercise-induced metabolome changes may help illustrate how different exercise modalities benefit T2DM patients.

After a 12-week 36-session exercise intervention, only FBG in the Tai Chi group showed a declining trend without statistical difference. However, the metabolic profiles changed in both serum and urine. Differential metabolites, post Tai Chi, were mainly involved in sphingolipid metabolism, primary bile acid biosynthesis, and BCAAs degradation, while those post walking were related to pathways including steroid hormone biosynthesis, phenylalanine metabolism, and arachidonic acid metabolism. After practicing Tai Chi, serum BCAAs declined, which was reported to be closely associated with IR and the future risk of metabolic and cardiovascular morbidities [57,58]. Studies have revealed that the gut microbiota of T2DM responders to exercise are associated with increased biosynthesis of short-chain fatty acids (SCFAs) and catabolism of BCAAs [59,60]. This indicates that Tai Chi may alleviate diabetes by enhancing BCAAs clearance metabolism in the body. In addition, serum metabolites in sphingolipid metabolism declined, whereas inosine in purine metabolism increased. Sphingolipids have been considered integral players in the development and pathogenesis of insulin resistance and T2DM, as well as emerging factors mediating glucotoxic and lipotoxic cell death [61]. Inhibition of de novo sphingolipid synthesis can deplete ceramides and improve mitochondrial function [62]. Inosine is reported to protect and reduce diabetes incidence in streptozotocin-induced diabetes and spontaneous diabetes in NOD mice [63], and alleviate diabetic peripheral neuropathy [64]. Increased 7alpha, 25-dihydroxy-4-cholesten-3-one and decreased taurine in primary bile acid biosynthesis were detected post Tai Chi. Enhanced intestinal bile acid signaling contributes to the metabolic benefits of glucose homeostasis, such as bile acid sequestration and bariatric surgery [65]. 7alpha, 25-dihydroxy-4-cholesten-3-one, a bile acid lipid molecule, is an intermediate product in the process of cholesterol transforming into primary bile acids. However, before secretion into the intestine, primary bile acids, including cholic acid and chenodeoxycholic acid conjugate with taurine, which protects against insulin resistance and diabetic complications [66], while lower levels of plasma phenylalanine and tyrosine are related to a higher risk of diabetic nephropathy in T2DM [67]. Urine vanillylamine and dopamine levels in the phenylalanine and tyrosine metabolism pathways increased after Tai Chi. Dopamine is a well-known neurotransmitter that is closely associated with diabetes. Dopamine agonists alleviate hyperglycemia in patients with diabetes, whereas their antagonists induce higher glucose and insulin secretion [68]. This could partly explain why exercise improved glucose control and pleasure. While after 12-week brisk walking, serum metabolites, including etiocholanedione, androstenedione, dehydroepiandrosterone, and corticosterone, in the steroid hormone biosynthesis pathway decreased. Steroids, also known as corticosteroids, can increase blood glucose levels. Metabolites of arachidonic acid metabolism were increased. Vascular prostacyclin synthesis is decreased in diabetic patients and experimental animals, yet insulin can reverse this situation [69].

Both Tai Chi and brisk walking can improve the metabolic health of T2DM patients to some extent. However, the two modalities seem to influence body metabolism in different ways. Unlike brisk walking, Tai Chi, a mind-body practice, integrates meditation and physical movement. It features *Tiaoxing* (physical regulation), *Tiaoshen* (mind regulation), and *Tiaoxi* (breath regulation), similar to other traditional Chinese exercises. Researchers are keen to investigate its working mechanism with the help of omics, gait analyzer [70], neuroimaging techniques such as fMRI [29], functional near-infrared spectroscopy (fNIRS) [71], pulmonary function detector [72] and other modern techniques, which differentiate it from other aerobic exercise modalities.

## Conclusion and study limitation

T2DM patients display impaired carbohydrate, lipid, and amino acid metabolism. Both Tai Chi and brisk walking can improve the metabolic health of T2DM patients to some extent. Tai Chi may improve disrupted BCAAs metabolism, whereas brisk walking may regulate steroid hormone biosynthesis and arachidonic acid metabolism.

Though as a pioneering study using untargeted metabolomics to investigate the effect of Tai Chi exercise on the metabolic profile of T2DM patients, the study results should be considered with the following limitations: small sample size, non-uniform accommodation, and a lack of controls of waiting-list and healthy individuals with Tai Chi. For a pilot omics study, the application of 10-case samples in similar omics studies [73–75] during that timeframe underline the relative adequacy of our sample size given the specific conditions. Additionally, we noted that recent high-quality omics studies on exercise intervention in diabetes, where sample sizes have expanded to 15–21 cases per group [76,77]. A sample size of 15–21 per group as reference to facilitate the scientific rigor and reliability of similar study in the future. As non-uniform accommodation (including diet and medication) may have influenced our findings, data collection on their dietary and medication use throughout the study duration, and subgroup analyses are needed to assess the potential impact of dietary and medication variations on the study outcomes in the future study. Besides, correlation between metabolomics and clinical outcomes was not done in this study, future studies to further verify the findings in both clinical and experimental settings. The future research endeavors will be taken to address these limitations by optimizing study design and implementation. Despite these limitations, this study has provided a reference for using metabolomics to investigate the mechanism of traditional exercise, and the evaluation of metabolomic profiling of individual patients can be used to design individualized diabetic treatments in the future.

## Supporting information

S1 Checklist CONSORT checklist. (DOCX)

S1 Fig PCA in serum and urine metabolomics between the T2DM and healthy controls. (TIF)

S2 Fig PCA in serum and urine metabolomics change of T2DM post and pre walking in positive ion mode. (TIF)

S3 Fig Volcano plots and PLS-DA in serum and urine metabolomics change of T2DM post and pre walking in positive ion mode. (TIF)

S1 Table Fasting blood glucose (FBG) and hemoglobinA1c (HbA1c) change in T2DM patients after Tai Chi and walking intervention. (DOCX)

S2 Table Identified differential metabolites in serum and urine of T2DM patients post and pre walking in positive (+) and negative (−) ion modes. (DOCX)

S1 File Research protocol (English). (PDF)

S2 File Research protocol (Chinese). (PDF)

S1 Data (XLSX)
