# Supplementary figures and images for: Metabolic impairments associated with type 2 diabetes mellitus and the potential effects of exercise therapy: An exploratory randomized trial based on untargeted metabolomics
Source: PLoS One. 2024 Mar 22;19(3):e0300593. doi: 10.1371/journal.pone.0300593 (PMC10959348; doi:10.1371/journal.pone.0300593)

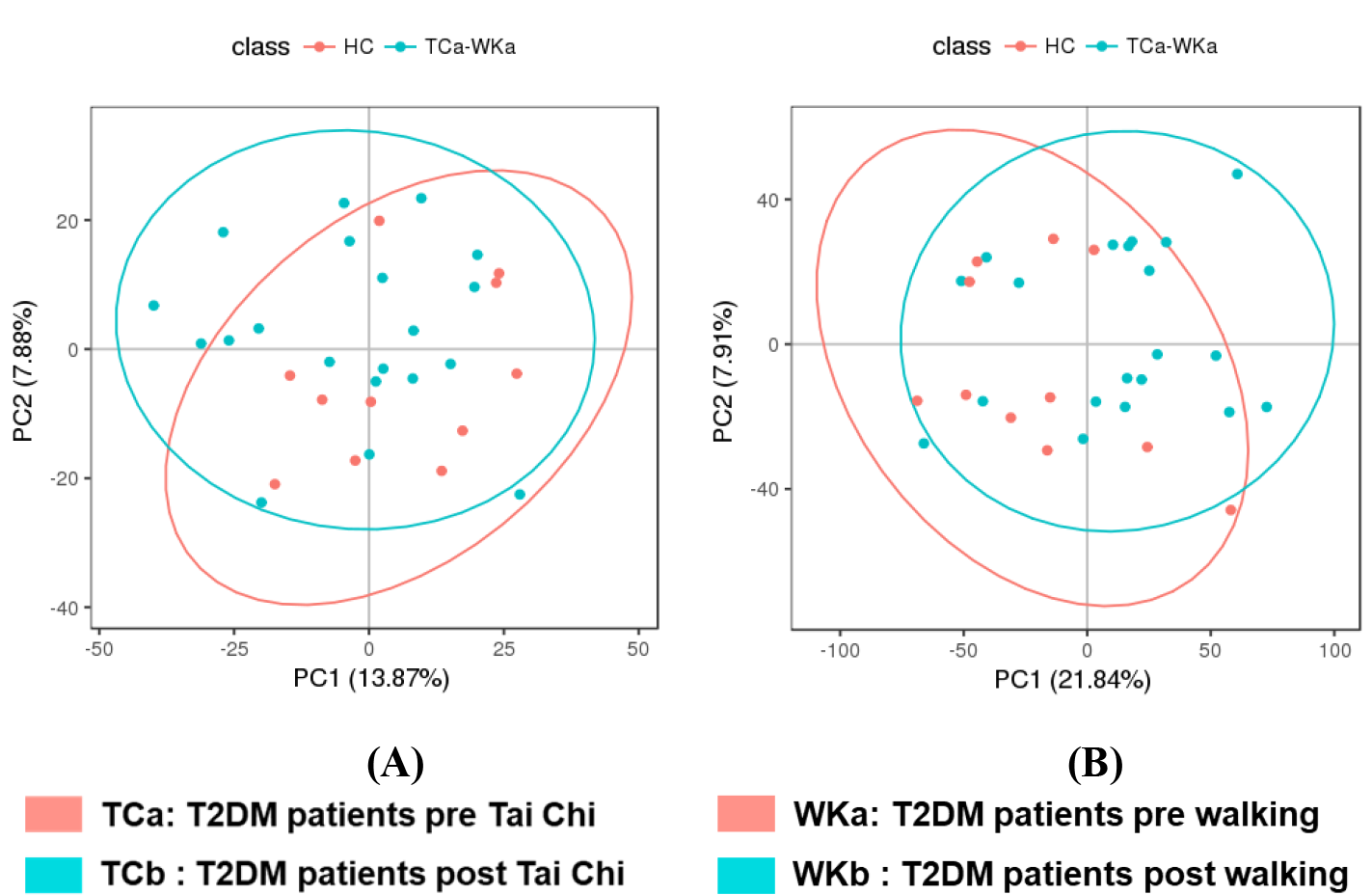

Supplement: S1 Fig — (TIF) [file pone.0300593.s002.tif]

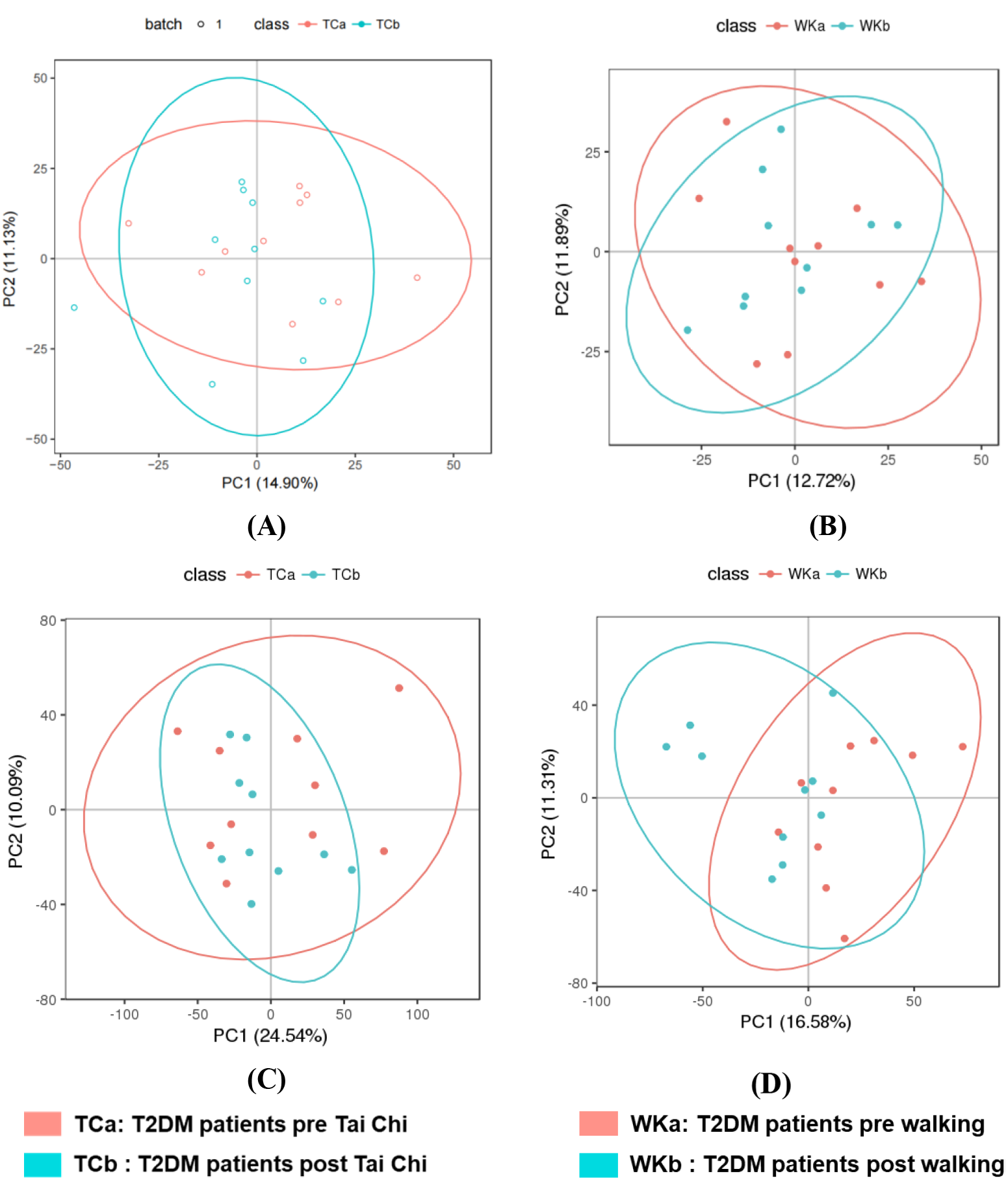

Supplement: S2 Fig — (TIF) [file pone.0300593.s003.tif]

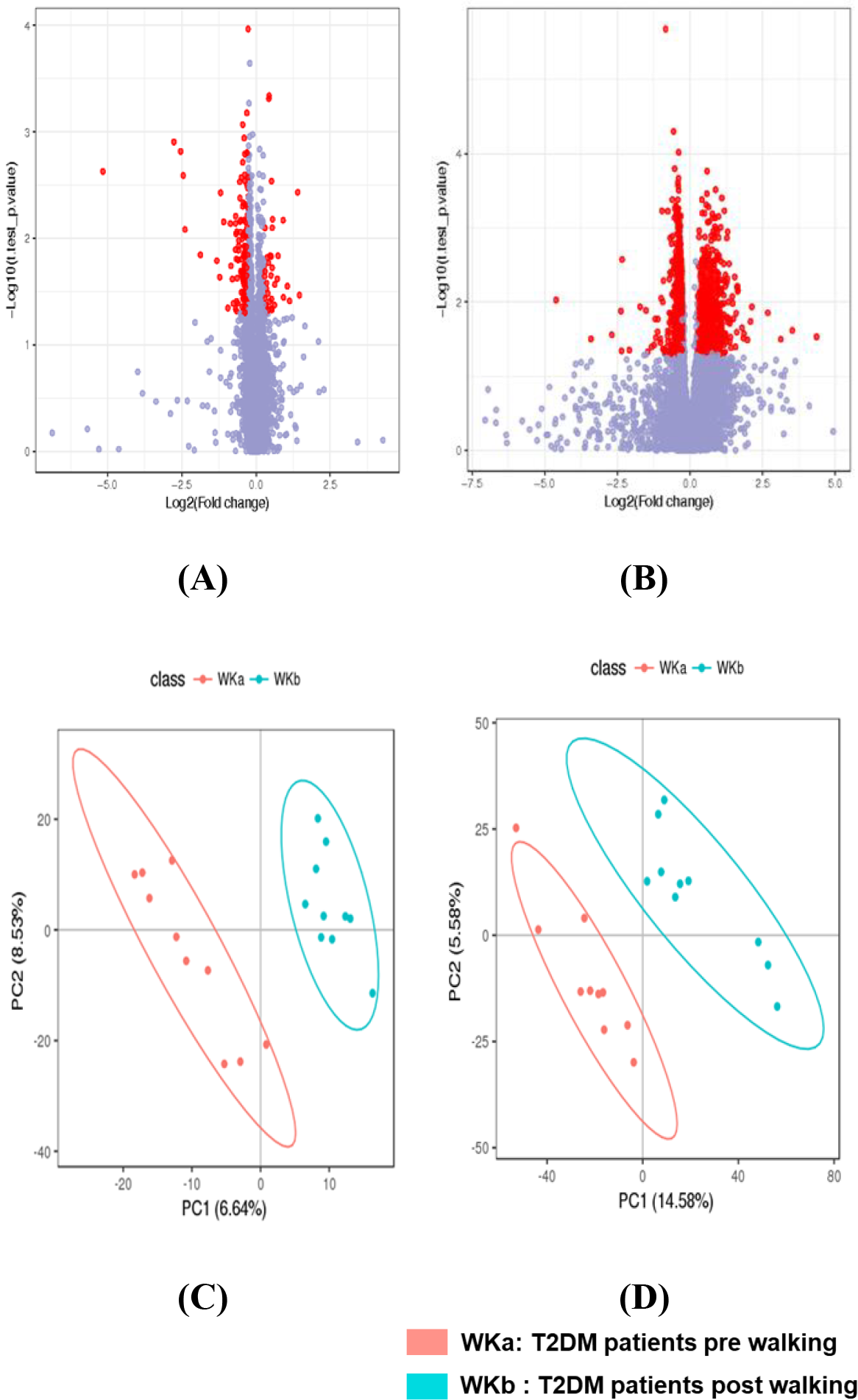

Supplement: S3 Fig — (TIF) [file pone.0300593.s004.tif]
