# Supplementary material for: Metabolic impairments associated with type 2 diabetes mellitus and the potential effects of exercise therapy: An exploratory randomized trial based on untargeted metabolomics
Source: PLoS One. 2024 Mar 22;19(3):e0300593. doi: 10.1371/journal.pone.0300593 (PMC10959348; doi:10.1371/journal.pone.0300593)
Supplement: S1 Table — (DOCX) [file pone.0300593.s005.docx]

**S1 Table. Fasting blood glucose (FBG) and hemoglobinA1c (HbA1c) change in T2DM patients after Tai Chi and walking intervention.**

| **Item** | **Tai Chi (n=10)** | | | |  | **Walking (n=10)** | | |  | **Intergroup p value** | |
| --- | --- | --- | --- | --- | --- | --- | --- | --- | --- | --- | --- |
|  | **pre** | | **post** | ***p* value** |  | **pre** | **post** | ***p* value** |  | **pre** | **post** |
| **FBG**  **(mmol/L)** | 7.94(6.51, 9.29) | 7.38(6.07, 7.78) | | 0.203 |  | 6.64(5.87, 8.25) | 6.75(6.05, 7.58) | 0.575 |  | 0.290 | 0.545 |
| **HbA1c** | 6.60(6.15, 8.03) | 6.75(6.05, 7.58) | | 0.386 |  | 6.25(5.80, 7.65) | 6.35(5.90, 7.18) | 0.507 |  | 0.544 | 0.448 |

Notes: data were expressed by median (Q1, Q3), Q1, first quartile; Q3, third quartile;
