# Supplementary material for: Metabolic impairments associated with type 2 diabetes mellitus and the potential effects of exercise therapy: An exploratory randomized trial based on untargeted metabolomics
Source: PLoS One. 2024 Mar 22;19(3):e0300593. doi: 10.1371/journal.pone.0300593 (PMC10959348; doi:10.1371/journal.pone.0300593)
Supplement: S2 Table — (DOCX) [file pone.0300593.s006.docx]

**S2 Table. Identified differential metabolites in serum and urine of T2DM patients post and pre walking in positive (+) and negative (−) ion modes**

| **Sample** | **No.** | **Compound ID** | **Trend** | **VIP** | **KEGG ID** | **Identification** | **pathway** |
| --- | --- | --- | --- | --- | --- | --- | --- |
| **Serum** | 1+ | 5.73_306.2435m/z | ↓ | 2.65 | C03772 | Etiocholanedione | Steroid hormone biosynthesis |
|  | 2+ | 5.73_306.2435m/z | ↓ | 2.65 | C00674 | Androstanedione |  |
|  | 3+ | 5.73_306.2435m/z | ↓ | 2.65 | C01227 | Dehydroepiandrosterone |  |
|  | 4+ | 0.65_347.2186m/z | ↓ | 6.52 | C02140 | Corticosterone |  |
|  | 5- | 4.22_178.0507m/z | ↓ | 2.58 | C05658 | Indoxyl | Tryptophan metabolism |
|  | 6- | 4.22_178.0507m/z | ↓ | 2.58 | C01586 | N-benzoylglycinate | Phenylalanine metabolism |
|  | 7- | 4.22_178.0507m/z | ↓ | 2.58 | C19569 | 3-Succinoylpyridine | Nicotinate and nicotinamide metabolism |
|  | 8- | 4.22_178.0507m/z | ↓ | 2.58 | C01586 | Hippuric acid | Phenylalanine metabolism |
|  | 9- | 7.42_313.2375m/z | ↓ | 2.22 | C14828 | 9,10-DHOME | Linoleic acid metabolism |
|  |  |  |  |  |  |  |  |
| **Urine** | 1- | 3.69_325.0919m/z | ↑ | 2.25 | C03406 | Argininosuccinic acid | Arginine biosynthesis |
|  | 2- | 5.79_367.2114m/z | ↑ | 2.03 | C05962 | 6-Ketoprostaglandin E1 | Arachidonic acid metabolism |
|  | 3- | 5.79_367.2114m/z | ↑ | 2.03 | C05956 | Prostaglandin G2 |  |
|  | 4- | 5.80_381.2250m/z | ↑ | 2.17 | C05356 | 5(S)-Hydroperoxyeicosatetraenoic acid |  |

T2DM patients pre and post walking in serum and urine metabolomics: p < 0.05, VIP > 2, FC >=1.2 or <=0.8333; +: positive ion mode; -: negative ion mode; ↑: up-regulated; ↓: down-regulated.
