# Supplementary material for: Metabolic impairments associated with type 2 diabetes mellitus and the potential effects of exercise therapy: An exploratory randomized trial based on untargeted metabolomics
Source: PLoS One. 2024 Mar 22;19(3):e0300593. doi: 10.1371/journal.pone.0300593 (PMC10959348; doi:10.1371/journal.pone.0300593)

Responsible Unit for the Project: Chengdu University of Traditional Chinese Medicine

Project Investigator: Rongjiang Jin

Version Number: VERSION 3.0

Version Date: 20180502

Impact of Tai Chi Exercise on Metabolomics in Type 2 Diabetes Patients

Chengdu University of Traditional Chinese Medicine

April 2018

CONTENTS

1. RESEARCH BACKGROUND 3

2. RESEARCH OBJECTIVES 3

3. RESEARCH CONTENT, RESEARCH METHODS, AND TECHNICAL ROUTE 4

3.1 Research Content 4

3.2 Research Methods 4

3.2.1 Subject Recruitment 4

3.2.2 Diagnostic Criteria 4

3.2.3 Inclusion Criteria 4

3.2.4 Exclusion Criteria 6

3.2.5Termination, Exclusion, and Dropout Criteria 6

3.2.6 Sample Size and Blinding 7

3.2.7 Grouping and Intervention Content 8

3.2.8 Biological Sample Collection 9

3.2.9 Outcome Assessment 9

3.2.10 Safety Evaluation 10

3.2.11 Statistical Analysis 11

3.2.12 Quality Control 11

3.2.13 Ethical Considerations 12

3.3 Study flowchart 14

Clinical Trial Research Protocol

1. Research Background

Diabetes mellitus (DM) has become the third most serious chronic non-communicable disease globally, following cardiovascular diseases and cancer, posing a significant threat to human health. According to epidemiological studies, the direct economic expenditure on diabetes worldwide exceeds 827 million US dollars annually. In China, the prevalence of DM is as high as 10.9%, with over 100 million diabetes patients, ranking first in the world, and related expenses amounting to 5.994 billion US dollars.

Traditional Tai Chi, a form of Chinese martial arts, is widely used in the prevention and treatment of diabetes and its complications. Preliminary clinical trial results from the research group indicate that Tai Chi has a good therapeutic effect on type 2 diabetes (T2DM) and its effects have a certain degree of sustainability. Other clinical research evidence also suggests that Tai Chi therapy for diabetes is affirmative, safe, and reliable, and clinical physicians are recommended to recommend it to T2DM patients for learning and exercise. However, its specific mechanism is not yet clear. Research has found that aerobic exercise can improve metabolic disorders in T2DM patients. Based on this, the following research hypothesis is proposed: The 24-form Tai Chi exercise may improve T2DM by affecting metabolism.

2. Research Objectives

To investigate the metabolic effects of the 24-form Tai Chi exercise intervention in patients with type 2 diabetes (T2DM) and to provide a microbiological basis for Tai Chi intervention in T2DM.

3. Research Content, Research Methods, and Technical Route

3.1 Research Content

Utilize non-targeted metabolomics techniques to study the impact of the simplified 24-form Tai Chi exercise on the metabolomics of patients with type 2 diabetes (T2DM).

3.2 Research Methods

3.2.1 Subject Recruitment

The subjects for this study will be recruited from T2DM patients at the affiliated hospitals of Chengdu University of Traditional Chinese Medicine and the Third Affiliated Hospital of Chengdu University of Traditional Chinese Medicine in Sichuan Province. Healthy volunteers will be recruited from the community in the same area.

3.2.2 Diagnostic Criteria

Based on the diagnostic criteria for T2DM outlined in the "Chinese Guidelines for the Prevention and Treatment of Type 2 Diabetes (2013 Edition)" issued by the Chinese Medical Association Diabetes Branch, patients meeting the criteria for clear diagnosis will be selected for the trial.

3.2.3 Inclusion Criteria

(1) Inclusion Criteria for T2DM Patients:

①Confirmed diagnosis of T2DM, with fasting blood glucose (FBS) > 7mmol/L and glycated hemoglobin (HbA1c) > 6.5%, using only oral hypoglycemic drugs without insulin treatment.

②Age between 45 and 75 years.

③Han nationality patients who have resided in Chengdu urban area for five years or more.

④No serious adverse habits such as chronic heavy smoking, alcohol abuse, drug abuse, etc.

⑤Relatively stable dietary habits without specific dietary preferences.

⑥Lack of regular exercise habits (less than 30 minutes of moderate to high-intensity ⑦exercise per week, General Self-Efficacy Scale (GSLTQ) score < 10), and no Tai Chi practice in the last three months.

⑧No use of antibiotics, gastrointestinal motility drugs, and microbiota-regulating agents or other drugs affecting the intestinal microbiota in the past month, and no gastrointestinal symptoms such as abdominal pain, constipation, and diarrhea.

⑨Voluntary participation in the study, obtaining permission from a specialized diabetes physician, and signing an informed consent form.

(Note: Only those meeting all the above conditions can be included.)

(2) Inclusion Criteria for Healthy Volunteers

Healthy individuals matching the age and gender of T2DM patients:

①Age between 45 and 75 years.

②Physically healthy with no history of endocrine or metabolic diseases, cardiovascular diseases, or other organ dysfunction.

③Lack of regular exercise habits.

④Han nationality individuals who have resided in Chengdu urban area for five years or more.

⑤No serious adverse habits such as chronic heavy smoking, alcohol or drug abuse, etc.

⑥Relatively stable dietary habits without specific dietary preferences.

⑦No use of antibiotics, gastrointestinal motility drugs, microbiota-regulating agents, or other drugs affecting the intestinal microbiota in the past month, and no gastrointestinal symptoms such as abdominal pain, constipation, and diarrhea.

3.2.4 Exclusion Criteria

①Individuals with other severe complications of diabetes, such as heart, brain, kidney, or eye complications.
②Those with a recent history of infection, surgery, or acute cardiovascular and cerebrovascular diseases.
③Individuals with a history of cancer.
④Those with severe liver or kidney dysfunction.
⑤Individuals with a history of prolonged use of antibiotics, hormones, or drug abuse.
⑥Individuals with other diseases affecting blood sugar, such as hyperthyroidism, Cushing's syndrome, etc.
⑦Individuals with severe hypertension (systolic blood pressure (SBP) > 160 mmHg, diastolic blood pressure (DBP) > 95 mmHg).
⑧Those with severe lower limb joint diseases and other conditions unsuitable for Tai Chi exercise.
⑧Individuals with a history of mental illness or a family history.
(Note: Individuals meeting any of the aforementioned conditions were not eligible for inclusion.)

3.2.5Termination, Exclusion, and Dropout Criteria

(1) **Termination Criteria**
①Participants experiencing severe adverse reactions during the study.
②Patients experiencing a worsening of their condition or other emergencies requiring urgent medical attention during the study.
③Participants with poor compliance, low participation, or inability to complete the research project.
④Use of antibiotics in the three months before sampling; intake of prebiotics or probiotic products in the last two weeks; a history of gastrointestinal diseases such as diarrhea or dysentery in the last month.

(2) Exclusion and Dropout Criteria

①Individuals mistakenly included who do not meet the inclusion criteria.

②Participants with poor compliance, uncooperative, or voluntary withdrawal during the study.

③Significant changes in the condition and routine treatment plan.

④Participants who do not undergo the scheduled examinations and follow-ups at the specified time.

⑤Individuals experiencing severe adverse events or complications that make it inadvisable to continue Tai Chi practice.

3.2.6 Sample Size and Blinding

Based on previous literature reports and expert recommendations, the sample size for each group is set at 12, considering a dropout rate of around 20%. With three groups in total, the total sample size for the study is 32, with 14 participants in each group.

The study will include outpatient T2DM patients at the affiliated hospitals of Chengdu University of Traditional Chinese Medicine and the Third Affiliated Hospital of Chengdu University of Traditional Chinese Medicine who meet the inclusion criteria from April 2018 to December 2019. They will be randomly divided into two groups. To ensure the concealment of the allocation scheme, before the start of the study, the trial designer will use the random number generator in SPSS version 20.0 to generate a random number sequence and load it into opaque envelopes after grouping. Patients who meet the inclusion and exclusion criteria and agree to participate in the study will be collected through outpatient visits. They will receive an opaque envelope in the order of their outpatient visits and will be assigned to a group based on the group information inside the envelope.

This study is an open-label study, and blinding is not implemented. However, a triple separation will be enforced for the interveners, data collectors, data entry personnel, and data analysts.

3.2.7 Grouping and Intervention Content

This trial is divided into three groups: Tai Chi Group, Walking Group, and Healthy Control Group. The treatment period is 12 weeks, with a follow-up after the end of the treatment for an additional 12 weeks.

(1) Tai Chi Group (TC)

Participants in the Tai Chi group will continue their routine medication and dietary habits while engaging in 12 weeks of continuous practice of the 24 simplified Tai Chi movements. The sessions will be conducted three times a week, with each session lasting 90 minutes, totaling 12 weeks. Professional Tai Chi instructors will provide on-site guidance, and each training session will follow a pattern of "20 minutes warm-up - 60 minutes Tai Chi practice - 10 minutes relaxation exercise." Participants will regularly attend diabetes patient health education activities, maintain a diabetes diary, and follow their normal lifestyle during the follow-up period, with information collected based on individual patient situations.

(2) Walking Group (WK)

Participants in the Walking group will maintain their routine medication and dietary habits unchanged while participating in 12 weeks of continuous walking exercise. The sessions will be conducted three times a week, with each session lasting 60 minutes, totaling 12 weeks. Participants will wear a Xiaomi wristband to monitor their walking steps. They will regularly attend diabetes patient health education activities, maintain a diabetes diary, and undergo scheduled tests and follow-ups.

(3) Healthy Control Group (HC)

Healthy volunteers in the control group will maintain their usual lifestyle and dietary habits, undergoing scheduled tests as required.

3.2.8 Biological Sample Collection

Participants in the Tai Chi group and Walking group, as well as healthy volunteers, will undergo biochemical blood tests, blood sample collection, and urine metabolic profiling before and after the treatment.

(1) Blood Sample Collection

Participants need to fast for at least 10 hours. A 5ml fasting venous blood sample will be collected for subsequent biochemical tests, including glycated hemoglobin, blood glucose, lipid profiles, and other biochemical indicators. Another venous whole blood sample will be centrifuged, and the serum will be collected and stored at -80°C.

(2) Urine Sample Collection

Midstream morning urine samples (20 ml) will be collected from the participants. After centrifugation, the samples will be transferred to 1.5ml cryovials and immediately stored at -80°C.

3.2.9 Outcome Assessment

(1) Biochemical Indicators:

Glycated hemoglobin

Fasting blood glucose

Lipid profiles (HDL, LDL, TG, TC)

Gastrin

(2) Metabolomic Indicators:

Major metabolites and their relative abundance in blood and urine

(3) General and Scale Indicators:

Body Mass Index (BMI)

Short Form-36 Health Survey (SF-36)

Borg Rating of Perceived Exertion (RPE)

3.2.10 Safety Evaluation

(1) Recording Adverse Events:

Record any adverse reactions (symptoms, signs) during the training process, including the time of occurrence, duration, severity, and actions taken. In the event of adverse reactions, the community physician and trial researchers should determine whether to terminate the trial based on the severity of the symptoms. Serious adverse events should be promptly reported to the project management center.

(2) Safety Controls for Tai Chi Training:

Participants must obtain permission from a specialized diabetes physician before participating in the study. Balance function testing will be conducted before and after training.

Researchers will observe participants throughout the exercise sessions. If abnormal symptoms such as pallor, profuse sweating, palpitations, or difficulty breathing occur, the exercise will be immediately stopped, and, if necessary, the participant will be sent for medical attention.

(3) Emergency Response Communication:

Through communication with community hospitals and patients' families, ensure that patients can receive emergency rescue in case of sudden dangerous situations during

exercise.

3.2.11 Statistical Analysis

(1) Clinical Data Analysis:

Clinical data will be presented as mean ± standard deviation. SPSS version 20.0 will be used for statistical analysis. The significance level (α) is set at 0.05, and P-values are two-tailed. A P-value less than 0.05 (two-tailed) is considered statistically significant.

Descriptive statistics: Frequency/percentage will be used for categorical data. For quantitative data, mean and standard deviation or median and quartiles (25th and 75th percentiles) will be used depending on the normality of the data.

Statistical analysis: One-way analysis of variance (ANOVA) will be used for quantitative data, and non-parametric tests will be used for categorical data.

(2) Metabolomic Data Analysis:

Univariate statistical analysis includes Student's t-test and fold-change analysis. R software will be used to create volcano plots.

Multivariate statistical analysis methods include unsupervised principal component analysis (PCA) and supervised partial least squares-discriminant analysis (PLS-DA) and orthogonal partial least squares-discriminant analysis (OPLS-DA).

3.2.12 Quality Control

(1) Design and Recruitment Phase:

Recruitment Phase Design: Develop a standardized implementation process and steps to ensure the standardization and consistency of the research protocol. Strictly adhere to inclusion and exclusion criteria for participant screening to ensure sample representativeness. Choose concise and targeted evaluation indicators suitable for participant characteristics. Utilize random group assignment of research subjects to

avoid bias. Explore and improve the feasibility of the research plan through the conduct of preliminary trials.

(2) Intervention Implementation Phase:

The researchers rigorously followed the research protocol to guide participants through Tai Chi exercise training, maintaining attendance records. Based on changes in participants' conditions, they provided guidance on lifestyle management or directed them to community hospitals when necessary. During the intervention, the research team communicated daily progress through the research center's WeChat group, held weekly discussion meetings to address encountered issues, and proposed corresponding solutions, ensuring the smooth progression of the study.

(3) Data and Record Management:

The research assistant employed a standardized questionnaire guide and filling method to instruct patients in completing the questionnaires. Throughout the entire study, both patients and research assistants remained unaware of the grouping conditions to reduce research contamination, information bias, and the Hawthorne effect. After data collection, a meticulous and timely verification process was implemented, addressing abnormal data and incomplete questionnaires by verifying and supplementing them promptly to ensure data completeness and accuracy. Data entry followed the principle of double entry by two individuals to guarantee data precision. Post data entry, random checks of 10% of the data, statistical descriptions to identify peculiar data, and logic corrections were employed to ensure data entry quality.

3.2.13 Ethical Considerations

(1) Adherence to Biomedical Ethics:

This study will strictly adhere to biomedical ethics principles. Before implementing the research, the research protocol and related documents, including the informed consent form, will be submitted to the ethics committee of the unit responsible for the project.

(2) Informed Consent:

Patients meeting the inclusion criteria will be informed about the purpose, process, and significance of the study. Their consent will be obtained in writing, and they will be informed that they can withdraw from the study at any time without affecting their rights, interests, or medical services.

(3) Data Confidentiality:

Strict adherence to the principle of data confidentiality will be maintained. Personal information of research participants will be kept strictly confidential. The data collected for the study will be used solely for research purposes and will not be used for any other purposes.

3.3 Study Flowchart

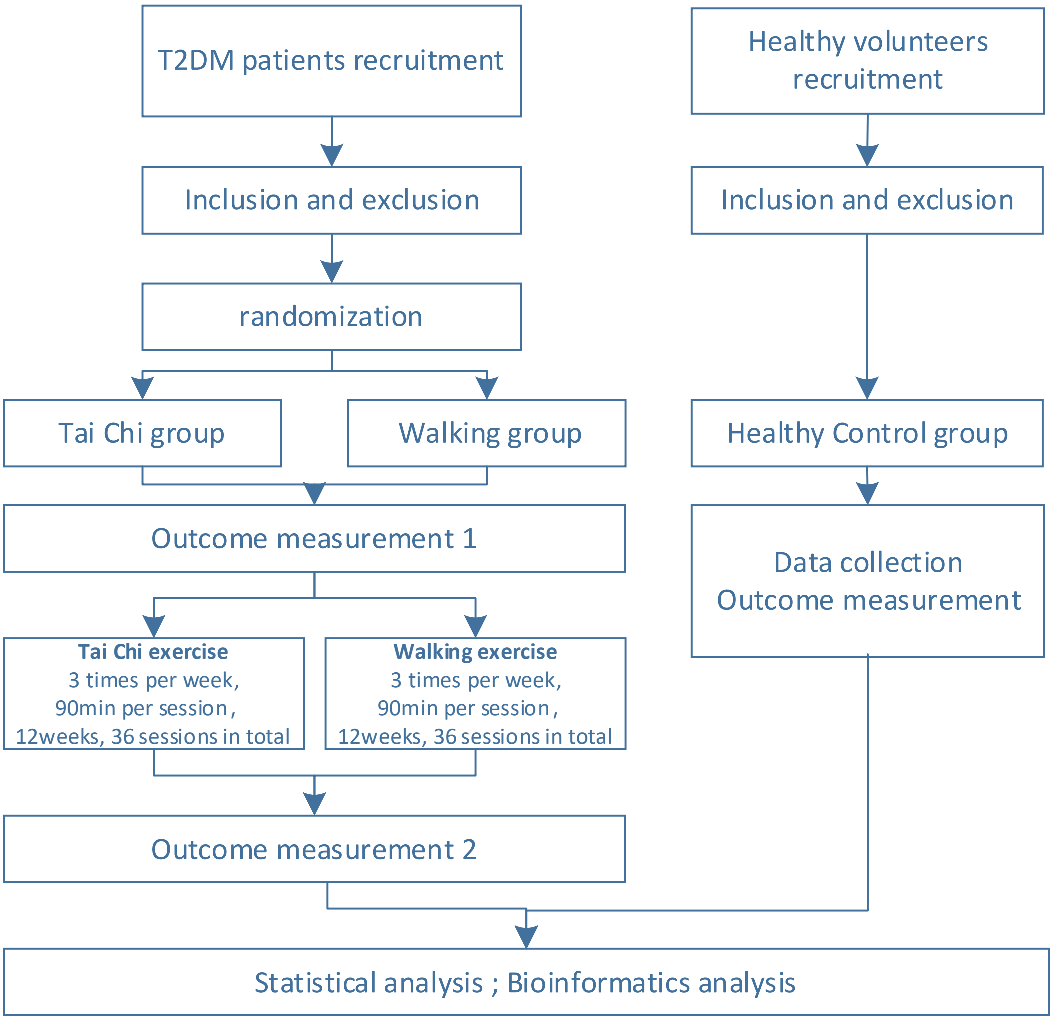

Supplement: S1 File — (PDF) [file pone.0300593.s007.pdf]
