# Supplementary material for: Metabolic impairments associated with type 2 diabetes mellitus and the potential effects of exercise therapy: An exploratory randomized trial based on untargeted metabolomics
Source: PLoS One. 2024 Mar 22;19(3):e0300593. doi: 10.1371/journal.pone.0300593 (PMC10959348; doi:10.1371/journal.pone.0300593)
Supplement: S2 File — (PDF) [file pone.0300593.s008.pdf]

课题负责单位：成都中医药大学

课题负责人：金荣疆

版本号：VERSION 3.0

版本日期：20180502

太极拳运动对 2 型糖尿病患者代谢组学的影响研究

2018 年 4 月

目录

1 研究背景 2

2 研究目的 2

3 研究内容、研究方法及技术路线........................ 2

3.1 研究内容... 2

3.2 研究方法... 3

3.2.1 受试者来源.................................... 3

3.2.2 诊断标准...................................... 3

3.2.3 纳入标准...................................... 3

3.2.4 排除标准...................................... 4

3.2.5 中止、剔除及脱落标准 4

3.2.6 样本量及盲法................................... 5

3.2.7 分组及干预内容 5

3.2.8 生物样本采集................................... 6

3.2.9 结局评估...................................... 6

3.2.10 安全性评价 6

3.2.11 统计分析..................................... 7

3.2.12 质量控制..................................... 7

3.2.13 伦理学问题.................................... 8

3.3 技术路线图....................................... 8

临床试验研究方案

1 研究背景

糖尿病（Diabetes melliltus, DM）已成为继心脑血管疾病、肿瘤之后全球第三大严重危害人类健康的慢性非传染性疾病。据流行病学研究显示[1-3]，全球每年糖尿病的直接经济支出超过8.27亿美元。中国DM发病率高达10.9%，已有超过1亿的糖尿病患者，位居世界第一，相关费用支出达到59.94亿美元[1]。

传统功法太极拳广泛用于糖尿病及其并发症的防治中，课题组前期临床试验结果表明太极拳对2型糖尿病（T2DM）具有良好疗效，且效应具有一定的持续性。其他临床研究证据[2-4]也表明太极拳治疗糖尿病疗效肯定，安全可靠，建议临床医师可以作为证据推荐给T2DM患者进行学习锻炼[4]。然而其具体机制尚不明确。研究发现有氧运动可以改善T2DM患者代谢紊乱，据此，提出如下研究假说：24式太极拳运动可能通过影响代谢改善T2DM。

2 研究目的

探讨 24 式太极拳运动干预对 2 型糖尿病(T2DM)患者代谢的影响，为太极拳干预 T2DM 提供微生物学依据。

3 研究内容、研究方法及技术路线

3.1 研究内容

运用非靶向代谢组学技术研究简化 24 式太极拳运动对 T2DM 患者代谢组学的影响。

3.2 研究方法

3.2.1 受试者来源

本研究受试者来源于四川省成都中医药大学附属医院、成都中医药大学第三附属医院的 T2DM 患者；健康志愿者来自于该片区的社区。

3.2.2 诊断标准

根据中华医学会糖尿病学分会颁布的《中国 2 型糖尿病防治指南（2013 年版）》中 T2DM 的诊断标准，筛选诊断明确并符合纳入标准的患者进行试验。

3.2.3 纳入标准

（1）T2DM 患者的纳入标准

① 确诊为 T2DM，空腹血糖 FBS＞7mmol/L，糖化血红蛋白 HbA1c＞6.5%，仅用口服降糖药，未使用胰岛素治疗者；

② 45 岁≤年龄≤75 岁；

③ 长期居住于成都市区的汉族患者（居住时间 5 年及以上）；

④ 无严重不良嗜好如长期大量吸烟，酗酒，吸毒等；

⑤ 饮食习惯相对稳定，无特别饮食偏嗜者；

⑥ 无规律运动习惯者（每周中高强度锻炼＜30 分钟，GSLTQ 量表评分＜10 分），近三个月未打太极拳者；

⑦ 近一个月未服用抗菌药物、胃肠动力药及微生态调节剂等影响肠道菌群的药物，且无腹痛、便秘及腹泻等胃肠道症状。

⑧ 自愿参加本研究，且获得糖尿病专科医师准许，签署知情同意书者；

（注：符合上述所有条件者才能纳入）

（2）健康志愿者的纳入标准

与 T2DM 患者年龄、性别相匹配的健康人

① 45 岁≤年龄≤75 岁；

② 身体健康无糖尿病等内分泌代谢病史、心脏病史或其他脏器功能异常

③ 无规律运动习惯者；

④ 长期居住于成都市区的汉族患者（居住时间 5 年及以上）；

⑤ 无严重不良嗜好如长期大量吸烟，酗酒，吸毒等；

⑥ 饮食习惯相对稳定，无特别饮食偏嗜者；

⑦ 近一个月未服用抗菌药物、胃肠动力药及微生态调节剂等影响肠道菌群的药物，且无腹痛、便秘及腹泻等胃肠道症状。

3.2.4 排除标准

① 伴有其他严重糖尿病心、脑、肾、眼等并发症者；

② 近期有感染、手术史、急性心脑血管疾病史者；

③ 肿瘤病史者；

④ 有严重肝肾功能损害者；

⑤ 长期服用抗生素、激素或有滥用药物史者；

⑥ 其他影响血糖的疾病史，如甲亢、库欣综合征等；

⑦ 严重高血压患者（收缩压（SBP）＞160 mmHg，舒张压（DBP）＞95 mmHg）；

⑧ 严重下肢关节疾病及其他不适宜太极拳运动者；

⑨ 精神疾病史及家族史者；

（注：凡具有上述任何一个条件者，均不能纳入）

3.2.5 中止、剔除及脱落标准

（1）中止标准

① 研究中出现严重不良反应者；

② 研究中出现患者病情加重或其他突发事件，需紧急就医者；

③ 患者依从性差，参与度过低，无法完成课题研究者；

④ 采样前 3 个月内使用过抗生素；近 2 周服用过益生元或者益生菌等制品；近 1 月有过腹泻、痢疾等胃肠道疾病史。

（2）剔除及脱落标准

① 不符合纳入标准，被误纳入者；

② 依从性差，不合作，中途自行退出者；

③ 病情及常规治疗方案发生重大改变者；

④ 未按规定时间进行检查、随访者；

⑤ 发生严重不良事件或并发症，不宜继续打拳者；

3.2.6 样本量及盲法

根据既往文献报道及专家建议，每组样本含量为 12 例，考虑 20%左右的样本脱失率，三组共纳入样本总量为 32 例，每组 14 例。

本研究将2018年4月至2019年12月四川省成都中医药大学附属医院、成都中医药大学第三附属医院符合纳入标准的门诊T2DM患者作为研究对象，随机分为两组。为了保证分配方案的隐藏，开始研究之前，试验设计者使用SPSS20.0版本统计分析软件的随机数字生成器产生随机数字序列，并分组后依次装入不透明信封内。通过门诊收集符合纳入排除标准并同意参与研究的患者，按照其门诊就诊的先后顺序依次获得一个不透明的信封，根据信封内的组别对患者进行分配。本研究属于开放性研究，故未设计盲法。但要求干预者、资料收集者、资料录入者和数据分析者实行三分离。

3.2.7 分组及干预内容

本试验共分三个组：太极拳组、步行组、健康对照组。治疗期12周，治疗结束后12周进行随访。

（1） 太极拳组（Taichi, TC）

太极拳受试者在保持常规用药和饮食习惯的同时，进行连续12周的24式简化太极拳运动，每周3次，每次90分钟，共12周。由专业太极拳老师进行现场教学指导，每次训练按照“热身运动20分钟 -打太极拳60分钟-放松运动10分钟”的模式进行锻炼。定期参加糖尿病患者健康宣教活动，记录糖尿病日记。随访期生活如常，并根据患者个人情况进行信息采集。

（2） 步行组（Walking，WK)

步行组的受试者在保持常规用药和饮食生活习惯不变，同时连续12周的步行运动，每周3次，每次60分钟，共12周，佩戴小米手环监测行走步数。 定期参加糖尿病患者健康宣教活动，记录糖尿病日记，并按时进行检测和随访。

（3） 健康人对照组(Healthy control，HC)

健康志愿者需保持平时的生活饮食习惯，按时进行检测。

3.2.8 生物样本采集

太极拳组、步行组受试者分别于治疗前后，健康志愿者在入组后进行血液生化检测、血液、尿液代谢组学检测。

（1）血样采集

受试者需空腹至少 10 小时。采集 5ml 空腹静脉血做后续的糖化血红蛋白、血糖、血脂等一系列生化指标检测；另一份静脉全血离心后取上清血清，冻存于-80℃。

（2）尿液采集

采集受试者中段晨尿 20 毫升，样本离心后分装至 1.5ml 的冻存管内，立即冻存于-80℃。

3.2.9 结局评估

（1） **生化指标**：糖化血红蛋白、空腹血糖、血脂指标（HDL，LDL，TG，TC）、胃泌素。

（2） **代谢组学指标**：血液、尿液主要代谢物及相对丰度。

（3） **一般及量表指标**：体重指数（BMI）、生活质量调查简表（SF-36）、伯格运动感觉量表(RPE)。

3.2.10 安全性评价

（1）记录训练过程中出现的不良反应（症状、体征），出现时间、持续时间、程度、处理措施等。发现不良反应时，社区医生与试验研究人员应依据病情程度判断是否中止试验，严重者及时上报课题管理中心。

（2）太极拳训练的安全控制：①受试者得到糖尿病专科医师的准许后方可参与本研究，另外在训练前后进行平衡功能测定。②研究者全程观察受试者在运动过程中的情况，若出现面色苍白、大汗、心悸、呼吸困难等异常症状，立即停止运动，必要时及时送医。

（3）通过与社区医院以及患者家属的沟通联系，确保患者在运动过程中突发危险情况，可以得到紧急救援。

3.2.11 统计分析

（1）临床数据用均数±标准差表示， 采用SPSS20.0统计软件处理，α值取0.05作为检验水准，P值为双侧概率。$P<0.05$ （双侧检验）认为具有统计学意义。统计描述：用频数/构成比描述计数资料，根据资料是否符合正态分布分别使用均数、标准差或中位数、四分位数（第25百分位数，第75百分位数）来描述计量资料。统计分析：计量资料采用单因素方差分析，计数资料采用非参数检验。

（2）代谢组学数据分析：维统计分析包括 Student’s t-test 和变异倍数分析，R 软件绘制火山图等；多变量统计分析方法包括无监督主成分分析（PCA）分析，有监督偏最小二乘法判别分析（PLS-DA）和正交偏最小二乘法判别分析（OPLS-DA）。

3.2.12 质量控制

（1）设计招募阶段：制定标准化的实施流程和步骤，保证研究方案实施的标准和一致；严格按照纳入和排除标准进行受试者的筛选，保证样本的代表性；选择条目精简、针对性明确的适合受试者特征的评价指标；采用随机分组分配研究对象，避免偏倚；通过预试验的开展对研究计划的可行性进行探索与改进。

（2）干预实施阶段：研究人员严格按照研究方案安排受试者进行太极拳运动训练，进行出勤登记，根据患者的情况变化指导其生活管理或社区医院就诊。在干预进行中，研究小组可以通过研究中心微信群及时汇报每天的工作进展，研究中心每周召开讨论组会，针对研究中遇到的问题进行商讨，并提出相应的解决方案，以保证研究的顺利进行。

（3）资料与数据管理：研究助理采用统一的问卷指导语与问卷填写方法指导患者填写问卷，在整个研究过程中，患者和研究助理对分组情况不知情，减少研究沾染、信息偏倚及霍桑效应。资料收集后，仔细、及时核查，对存在异常数据和遗漏的不完整问卷进行核实补填，保证数据完整和真实。采用双人双录的原则进行数据录入，保证资料准确性。资料录入后，采取随机复查 10%数据、统计描述发现奇异数据、逻辑纠错等方法保证录入质量。

3.2.13 伦理学问题

(1) 本研究将严格遵循生物医学伦理守则，在研究实施前将研究方案和知情同意书等相关研究资料送课题承担单位伦理委员会提出申请。

(2) 向符合纳入标准的患者介绍研究的目的、过程及意义，征得其同意并签署书面知情同意书，并告知研究对象可在任何时候退出研究且不影响其权益和利益，也不影响其接受医疗服务。

(3) 严格遵守研究资料保密原则，对参与研究的调查对象个人资料予以严格保密，研究收集的资料均只用于本研究，不做他用。

3.3 技术路线图

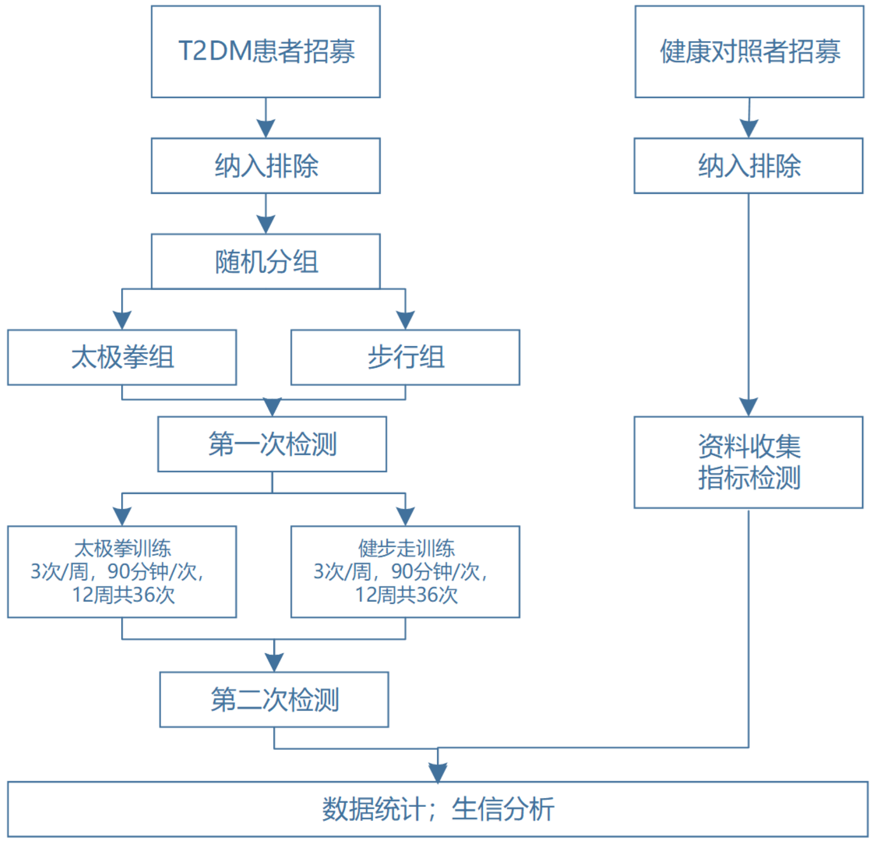

参考文献

[1] Whiting D R, Guariguata L, Weil C, et al. IDF diabetes atlas: global estimates of the prevalence of diabetes for 2011 and 2030.[J]. Diabetes Research & Clinical Practice, 2011, 94(3):311-321.

[2] Lancet. Worldwide trends in diabetes since 1980: a pooled analysis of 751 population-based studies with 4.4 million participants.[J]. Lancet, 2016, 387(10027):1513.

[3] Ogurtsova K, Da R F J, Huang Y, et al. IDF Diabetes Atlas: Global estimates for the prevalence of diabetes for 2015 and 2040[J]. Diabetes Res Clin Pract, 2017, 128:40-50.

[4] Huston P, Mcfarlane B. Health benefits of tai chi: What is the evidence?[J]. Canadian Family Physician, 2016, 62(11):881-890.
